# Thermodynamic Selection of Steric Zipper Patterns in the Amyloid Cross-*β* Spine

**DOI:** 10.1371/journal.pcbi.1000492

**Published:** 2009-09-04

**Authors:** Jiyong Park, Byungnam Kahng, Wonmuk Hwang

**Affiliations:** 1 Department of Physics and Astronomy, Seoul National University, Seoul, Korea; 2 Department of Biomedical Engineering, Texas A&M University, College Station, Texas, United States of America; National Cancer Institute, United States of America and Tel Aviv University, Israel

## Abstract

At the core of amyloid fibrils is the cross-*β* spine, a long tape of *β*-sheets formed by the constituent proteins. Recent high-resolution x-ray studies show that the unit of this filamentous structure is a *β*-sheet bilayer with side chains within the bilayer forming a tightly interdigitating “steric zipper” interface. However, for a given peptide, different bilayer patterns are possible, and no quantitative explanation exists regarding which pattern is selected or under what condition there can be more than one pattern observed, exhibiting molecular polymorphism. We address the structural selection mechanism by performing molecular dynamics simulations to calculate the free energy of incorporating a peptide monomer into a *β*-sheet bilayer. We test filaments formed by several types of peptides including GNNQQNY, NNQQ, VEALYL, KLVFFAE and STVIIE, and find that the patterns with the lowest binding free energy correspond to available atomistic structures with high accuracy. Molecular polymorphism, as exhibited by NNQQ, is likely because there are more than one most stable structures whose binding free energies differ by less than the thermal energy. Detailed analysis of individual energy terms reveals that these short peptides are not strained nor do they lose much conformational entropy upon incorporating into a *β*-sheet bilayer. The selection of a bilayer pattern is determined mainly by the van der Waals and hydrophobic forces as a quantitative measure of shape complementarity among side chains between the *β*-sheets. The requirement for self-complementary steric zipper formation supports that amyloid fibrils form more easily among similar or same sequences, and it also makes parallel *β*-sheets generally preferred over anti-parallel ones. But the presence of charged side chains appears to kinetically drive anti-parallel *β*-sheets to form at early stages of assembly, after which the bilayer formation is likely driven by energetics.

## Author Summary

Accumulation of amyloid fibrils is a salient feature of various protein misfolding diseases. Recent advances in precision experiments have begun to reveal their atomistic structures. Quantitative elucidation of how the observed structures are selected over other possible filament patterns would provide much insight into the formation and properties of amyloid fibrils. Using computer simulations and structural modeling, we demonstrate that the most stable filament pattern corresponds to the experimentally observed structure, and molecular polymorphism, selection of two or more patterns, is possible when there are more than one most stable structures. Ability to predict the structure allows for more detailed analysis, so that, for example, we can identify the most important residue for stabilizing the structure that could be therapeutically targeted. Our analysis will be useful for comparing different amyloid structures formed by the same protein or when delineating roles of different intermolecular forces in filament formation.

## Introduction

Amyloid fibrils are hallmarks of several neurodegenerative diseases including Alzheimer's, Parkinson's, and prion diseases [1]. Unlike other protein quaternary structures [2], amyloid fibrils share a sequence independent structural motif known as the cross- *β* spine; individual strands from constituent proteins forming a *β*-sheet that runs perpendicular to the fibril axis [3]. Amyloid fibrillogenesis is a multi-staged protein aggregation process and accumulating evidence suggests that prefibrillar oligomeric species are toxic [4]. Yet pathological roles of fibrillar species cannot be undermined. Amyloid protofibrils as well as oligomers have been suggested to lead to neuronal cell death [5]–[8]. Interruption of fibril formation prevented cell damage [9], and *β*-sheet rich diffusible oligomeric species of A *β*, the chief constituent of amyloid fibrils in Alzheimer's disease, possess cytotoxicity, which share structural similarity to mature fibrils [10]. In the case of systemic amyloidosis, sheer amount of amyloid deposition itself can be symptomatic [11]. Recent findings suggest even greater biological role of amyloid fibrils: amyloid fibrils in semen accelerated HIV infection [12]; a functional, mammalian amyloid composed of a protein Pmel17 promoted the formation of melanin [13]. Furthermore, *de novo* designed peptides self-assemble into amyloid-like *β*-sheet filaments, and hydrogels composed of these filaments hold a great potential for three-dimensional cell culture scaffold [14],[15].

Amyloid fibrils can be formed by a wide variety of protein sequences, where partial denaturation is a common precursor to fibril formation [16]. Evolution appears to have limited protein sequences in a restricted range of physico-chemical properties, *i.e.* in hydrophobicity and net electrostatic charges, to keep proteins from misfolding and aggregation [17]. Molecular polymorphism is another feature of amyloid fibrillogenesis, where a given peptide or protein may self-assemble into filament structures that differ in atomistic order as well as in filament morphologies [18]. While the selection of the filament structure depends on the growth condition, which can be purely mechanical agitation, once a stable filament is formed, it continues to grow, keeping the atomistic order even if the growth condition changes [19],[20].

The selection mechanism for the cross-*β* structure of amyloid fibrils is yet to be elucidated. Previous experimental approaches such as x-ray fiber diffraction [21], solid-state nuclear magnetic resonance (NMR) [22],[23], atomic force microscopy (AFM) [24], and electron microscopy (EM) [25] have contributed greatly to understanding molecular structures of amyloid fibrils as well as gross fibril morphology. More recently, x-ray diffraction of amyloid microcrystals enabled unequivocal determination of high-resolution atomistic structures of the cross-*β* spine [26],[27]. These results suggested that cross-*β* spines share a common structural feature termed as the ‘steric zipper,’ where side chains from the two *β*-sheets form a tightly interdigitating dehydrated interface, so that the resulting *β*-sheet bilayer forms a fundamental building block of fibrillar aggregates.

While these experiments are essential for describing supramolecular structures of amyloid fibrils, a fundamental question remains regarding how these structures are formed. Knowledge of the assembly pathway and structural properties of these fibrils would be useful for developing therapeutic strategies against amyloidoses as well as for developing biomaterials based on peptide self-assembly into *β*-sheet fibrils. Computer simulations have played an important role in addressing these questions. The assembly kinetics of *β*-sheet rich oligomeric species was characterized by the initial hydrophobic collapse followed by reorganization of monomers to form backbone hydrogen bonds [28],[29]. The potential of mean force of peptide dimers was calculated [30],[31]. Aggregation prone spots in an amyloidogenic protein were identified by dividing the protein into segments and performing simulations on each [32]. Relative stability of oligomers as well as mature filaments were also studied [33],[34]. More recently, various interaction modes between two *β*-sheets formed by human Islet amyloid polypeptides were studied [35].

Properties of high-resolution x-ray structures of cross-*β* spines [26],[27] have also been studied computationally. Molecular dynamics (MD) simulations of isolated *β*-sheet bilayer filaments showed stability of the steric zipper while the filament developed a helical twist [36]. The stability of spontaneously formed oligomers as well as oligomeric segments of the filament has been tested [37]–[39]. A thorough structural analysis on various oligomeric *β*-sheet species addressed a possible toxicity mechanism via non-zipper type exposed strands [40]. An *ab initio* quantum mechanical as well as classical electrostatics calculation showed that energetics of *β*-sheet formation is cooperative up to the length of three peptides [41]. In addition to simulations of available structures, knowledge-based modeling technique exploiting the crystallographic structures was developed to identify fibril-forming segments of proteins [42] and filament symmetry was utilized to predict detailed *β*-sheet bilayer conformation [43]. Possible binding modes of typical amyloid markers, congo red and thioflavin-T, on these fibrils were also studied computationally [44],[45].

Despite these advances in structural characterization, a basic question remains regarding the selection mechanism for steric zipper patterns. For a given peptide sequence, there are multiple ways of constructing *β*-sheets and stacking them [46],[47]. The 13 available crystal structures of the cross-*β* spine (‘steric zipper’) are classified into 8 different patterns depending on, 1) the relative direction of successive peptides in each layer, 2) the choice of the face of the *β*-sheet making the dehydrated interface, and 3) the symmetry between adjacent *β*-sheet layers [27]. Yet it is unclear how a given peptide in this study ended in a specific bilayer pattern. Although it is expected that the crystal structure corresponds to a free energy minimum among possible filament patterns, no quantitative study exists to demonstrate this to date.

Molecular polymorphism in amyloid fibrils as mentioned above further complicates the picture, where factors such as mechanical agitation [19] or different ionic strengths [22],[48] can lead to different supramolecular structures (reviewed in [18]). As a related issue, one of us found that at early stages of assembly, kinetic trapping may dominate over free energy minimization, as the conformational relaxation time for kinetically trapped oligomers is longer than the diffusional encounter time with other monomers and oligomers, supporting the possibility that kinetically trapped structure can propagate into the filament level [28]. Indeed, there are two filament structures for the peptide NNQQ, Protein Data Bank (PDB) IDs 2ONX and 2OLX, where their crystallization conditions differ only in the contents of the reservoir solution. It has been suggested that polymorphism is possible if there are multiple filament structures with similar thermodynamic stability [18]. However no quantitative study is available that supports this picture. It is also unclear whether similar stability between multiple possible filament structures is a requirement for polymorphism, or whether a filament structure can be chosen by a purely kinetic mechanism even if it is not the most stable one (with a free energy barrier with the most stable one being sufficiently larger than 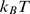, where 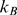: Boltzmann constant, *T*: temperature). It thus appears that, although amyloid fibrillogenesis is largely a sequence-independent phenomenon, for a given peptide sequence, the choice of specific steric zipper pattern involves intricate interactions between amino acid side chains as well as backbone hydrogen bonds. A comprehensive method that elucidates the structural selection mechanism and the stabilizing role of individual residues in the filament is thus desirable.

To address these issues, we adopted methods for calculating protein-protein binding energies [49]–[51] into a computational modeling and simulation scheme that calculates the *binding free energy* (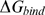) of a monomer incorporating into a given steric zipper pattern. It employs explicit water simulations to generate the coordinate trajectory and then uses a molecular mechanics/generalized Born-surface area method [52] and normal mode analysis (NMA) [53] to calculate various energy terms in 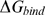. We constructed a series of steric zipper patterns for a given peptide and calculated 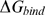 for each of them.

Our results quantitatively support the qualitative argument suggested previously: the minimum free energy configuration corresponds to the native steric zipper pattern found in x-ray crystallography, and molecular polymorphism is possible when there exist similarly stable filament patterns. Furthermore, detailed characterization of individual energy terms allowed to identify key interactions driving the bilayer formation: van der Waals (Lennard-Jones) and hydrophobic interactions contribute the most to the selection and stabilization of steric zipper patterns. Key residues in a given peptide sequence contributing the most to 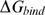 were identified to be those buried in the dehydrated interface between two *β*-sheets, suggesting the importance of tight side chain packing at the interface. Once a *β*-sheet is formed, shape complementarity is the major factor determining the bilayer pattern. But we found that formation of a *β*-sheet type is more prone to be affected by kinetic factors. In particular, for short peptides, charged side chains change the preference from parallel to anti-parallel *β*-sheets, which is not necessarily energetically favorable for steric zipper formation. As the most stable filament patterns identified through our method aligned well with the corresponding x-ray structures, in addition to detailed characterization of energetics, our analysis opens the possibility of predicting the cross-*β* spine structure and polymorphism formed by short peptides in atomistic accuracy.

## Results

**Figure 1:**
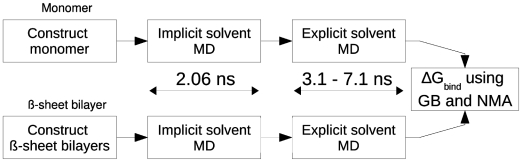
Overview of the simulation and analysis procedure. Starting from a peptide monomer, we constructed candidate *β*-sheet bilayer patterns. Molecular dynamics (MD) simulation of both the monomer and bilayers were performed first in implicit solvent to relax the initial structures then in explicit water for accurate trajectory generation. The first 1.1 ns of the explicit water simulation was the heating and equilibration phase. The remaining production run at 300 K lasting 2 to 6 ns was used to calculate ΔG*_bind_* via a generalized Born (GB) solvation model [52] and normal mode analysis (NMA) [53]. The long preparatory runs in the implicit solvent and the 1-ns equilibration in explicit water drove most bilayer patterns into fairly stable states, so that the profile of ΔG*_bind_* did not vary greatly throughout the production run, which was more prominent for native-like structures (cf., Figs. S1 and S4–S8).

We tested five different peptide sequences: 1) GNNQQNY from yeast prion Sup35, 2) NNQQ, a shorter derivative of GNNQQNY, 3) VEALYL from insulin, 4) KLVFFAE from 16th–22nd residue segment of the A *β* peptide, and 5) STVIIE, a *de novo* designed segment forming amyloid filaments [54] (Table 1). X-ray structures exist for the first three, with PDB IDs: 1YJP (GNNQQNY), 2ONX and 2OLX (NNQQ), and 2OMQ (VEALYL) [26],[27]. There is a solid state NMR structure for the KLVFFAE filament [22]. On the other hand, there is no available experiment determining the atomistic structure of the STVIIE filament, while simulation indicates a preference for anti-parallel *β*-sheet [54]. Filaments made of these peptides include both parallel and anti-parallel *β*-sheets, and they have different steric zipper patterns. Thus they cover a good spectrum of filament structures. We applied the procedure outlined in Fig. 1 to these systems. Note that we did not use x-ray structures for modeling and simulation, but explicit calculation of the binding free energy allowed us to select the most stable *β*-sheet bilayer patterns among those tested, which corresponded to x-ray structures fairly accurately.

**Table 1:** Summary of simulated configurations.

Peptide	Orie.	pH	*d* (Å)	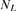
GNNQQNY	P	2.0	4.87 (4), FBC (2)	6
GNNQQNY	P	2.0	4.87 (2)	10
GNNAQNY	P	2.0	4.87 (2)	6
NNQQ	P	7.0	4.85 (2–4), 4.92 (2–4), adj. (2)	6
VEALYL	A	7.0	4.85 (4–6)	6
KLVFFAE	A/P	7.0	adj. (4)	6
KLVFFAE	A	7.0	adj. (4)	10
KLVFFAE	A	2.0	adj. (4)	6
STVIIE	A	7.0	adj. (2–4)	6

Orie.: Orientation, P/A: Parallel/Anti-parallel *β*-sheets. The protonation status of titratable groups at termini and charged side chains was determined based on pH in the experimental condition. The inter peptide distance (*d*) was either fixed or adjusted (adj.) using a constant pressure method. FBC: Free (not periodic) boundary condition. Numbers in parentheses after *d* are lengths of explicit-water production runs in nanoseconds. For some peptides, simulation times vary among different patterns, thus are given as ranges. Although the selection for the most stable bilayer patterns became clear usually within the first 2 ns of the production run (except for KLVFFAE at pH 7.0), a more converged profile of 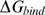 required longer simulation time, as explained in the text, and in Figs. 1, S1, and S4–S8. Thus we extended simulation time for selected (NNQQ, VEALYL,and STVIIE) or all (GNNQQNY and KLVFFAE) bilayer patterns. 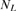: the number of peptides in a single *β*-sheet. Considering multiple bilayer patterns tested for each peptide, the total length of production runs in explicit water was over 290 ns.

For free energy calculation, we consider only states before and after association [49]–[51], rather than considering the reaction coordinate involved in the association process. This approach is computationally efficient and useful for studying macromolecular assemblies, compared to other more expensive methods [55]. The method combines explicit water simulation for generating faithful structures, and the Generalized Born with a simple SWitching (GBSW) continuum solvent model [52] for efficient initial relaxation of the structure and for calculating the solvation free energy from the coordinate trajectory of the explicit water simulation (Fig. 1; see Methods). Entropic contribution from vibrational modes of the molecule was calculated using NMA [56].

### Possible 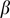-sheet bilayer patterns

GNNQQNY and NNQQ form parallel *β*-sheets while VEALYL, KLVFFAE, and STVIIE form anti-parallel *β*-sheets. As experiments indicated that a *β*-sheet bilayer with a dehydrated interface between the two sheets is the basic building block of filamentous aggregates [26],[27], we considered possible bilayer patterns formed by two identical *β*-sheets.

**Figure 2:**
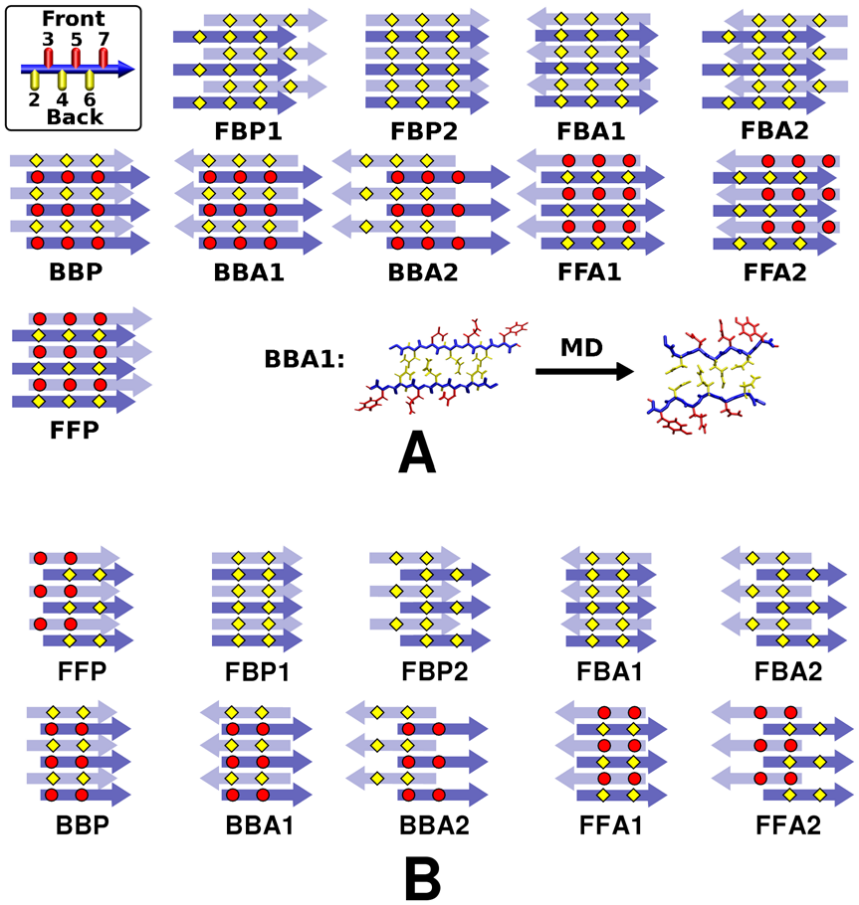
Ten possible *β*-sheet bilayer patterns of parallel *β*-sheets. (A) GNNQQNY and (B) NNQQ. The filament axis is vertical, and top/bottom layers are represented by dark/light arrows, where each arrow represents a single peptide. Top left in (A): A side view of a single GNNQQNY peptide with even-/odd-numbered side chains in yellow/red, which defines Front/Back faces of the parallel *β*-sheet. Bottom right in (A): relaxation of BBA1 after MD (axial view; cf. Fig. 5).

In the case of parallel *β*-sheet bilayers, we constructed ten possible patterns (Fig. 2). Naming schemes for these filaments are: **F**/**B** (front/back): even- (front) or odd-numbered (back) side chains buried in the bilayer, **P**/**A** (parallel/anti-parallel): relative direction between peptides in the two sheets. **1**/**2**: two choices of side-chain registry in the steric zipper. FFP and BBP did not have 1 or 2 due to rotational symmetry with respect to the filament axis.

**Figure 3:**
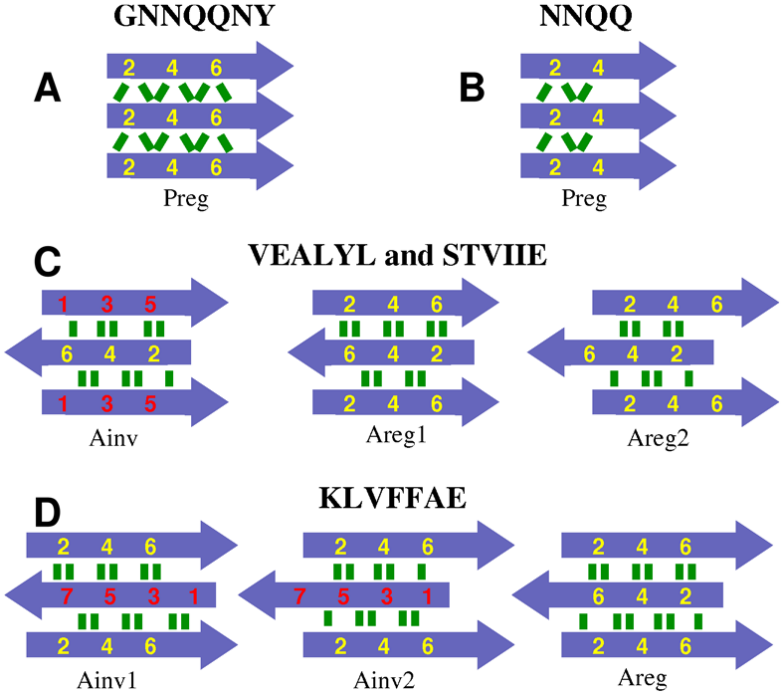
Peptide registry in a single *β*-sheet considered in this study. Parallel in-register (Preg) pattern maximizes the number of backbone hydrogen bonds (green lines) in (A) and (B). There are comparable numbers of backbone hydrogen bonds in the anti-parallel *β*-sheets shown in (C) and (D). Color codes are the same as in Fig. 2.

**Figure 4:**
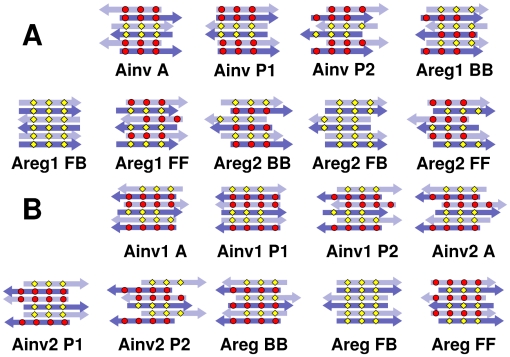
Nine possible configurations of anti-parallel *β*-sheets. Depending on the number of amino acids, there were distinct sets; (A) VEALYL and STVIIE, and (B) KLVFFAE. Arrows and color codes are the same as in Fig. 2.

Similarly, we constructed nine candidate bilayer patterns formed by two identical anti-parallel *β*-sheets (Figs. 3 and 4). For a single anti-parallel *β*-sheet, there are two possibilities, either face of a *β*-sheet composed entirely of even- or odd-numbered residues (**Areg**) or they alternate and appear on both faces (**Ainv**) [28]. VEALYL (KLVFFAE) has two possible Areg (Ainv) patterns with comparable number of backbone hydrogen bonds between neighboring peptides within a *β*-sheet, which we distinguish by **1** and **2** (Fig. 3). So there are *β*-sheet patterns of Ainv, Areg1, and Areg2 for VEALYL, and Ainv1, Ainv2, and Areg for KLVFFAE. In forming a bilayer, an Ainv pattern can be either symmetric (**P**) or anti-symmetric (**A**) against 180° rotation along the filament axis. Furthermore, as in parallel *β*-sheet, there are two choices of side chain registry in the dehydrated interface of an AinvP bilayer, **1** and **2**.

One potential problem with constructing *β*-sheet bilayer filaments is in side chain orientations. Once a peptide was within a *β*-sheet, its side chains did not easily rotate in simulations at 300 K, especially those buried in the dehydrated interface. This makes it difficult for the side chains to find proper orientations. For example, in the 1YJP structure of GNNQQNY, there are lateral hydrogen bonds through N2, Q4, and N6 in the dehydrated interface. At a higher temperature, however, side chains readily rotated to find their native-like orientations. We tested this by constructing a small BBA1 bilayer composed of 2 GNNQQNY peptides on each sheet, and performed a 2.5-ns simulation in implicit solvent at 350 K under a periodic boundary condition (PBC; see Methods). Side chains buried within the bilayer rotated readily to form proper hydrogen bonds. Since amyloidogenic peptides generally have polar side chains, it is thus desirable to first perform a quick high temperature relaxation run for a small *β*-sheet bilayer and construct larger systems using the relaxed structure.

Note that the 10 parallel and 9 anti-parallel *β*-sheet bilayer patterns that we considered do not exhaustively include all possibilities. However, they represent major sets of likely *β*-sheet bilayers in terms of side chain packing and backbone hydrogen bonding. These patterns cover eight classes of steric-zipper patterns proposed by Sawaya, et al. [27] (Table S1).

### Minimum free energy configuration of GNNQQNY corresponds to the x-ray structure

**Figure 5:**
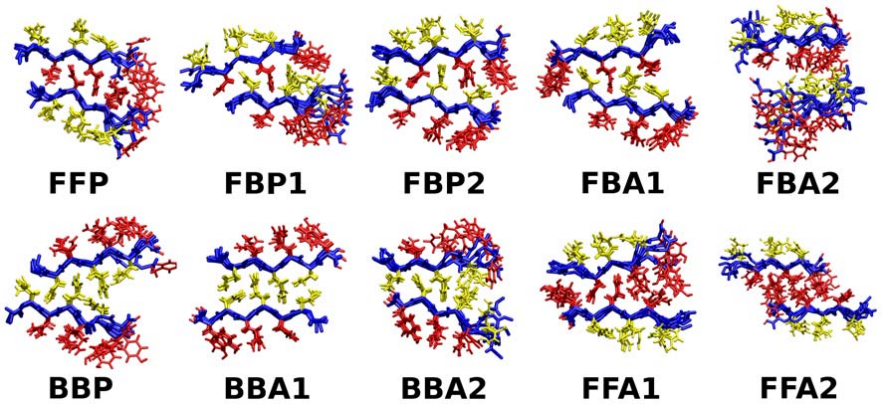
Conformations of candidate bilayer patterns of GNNQQNY after MD (axial view). Snapshots at 2 ns of the production run was used to draw each figure. Color scheme is the same as in Fig. 2.

**Figure 6:**
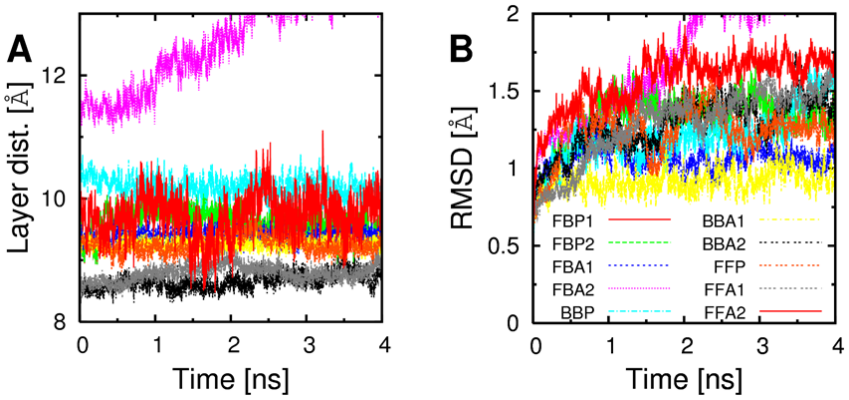
Integrity of *β*-sheet bilayer patterns during the production run. (A) Distance between *β*-sheets in a bilayer. (B) RMSD of C*_α_* atoms from the first snapshot. Within the simulation time, no filament except for FBA2 dissociated. The distance between *β*-sheets was defined by the distance between the least-squares-fit plane spanned by C*_α_* atoms in one layer and the center of mass of C*_α_* atoms in the other layer.

After constructing the bilayer, peptides were in extended conformations, which subsequently relaxed during MD simulations ( *e.g.*, Fig. 2A, bottom; Fig. 5. During 4-ns production runs of GNNQQNY bilayers, nearly all patterns maintained the integrity of *β*-sheets and dehydrated interfaces: the inter-layer distance fluctuated at most by 0.25 Å except for FBA2 whose sheets separated, and FBP1 that showed a significant fluctuation (Fig. 6A). For those that had small root-mean-square deviation (RMSD) of 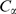 atoms from the structure at the beginning of the production run (Fig. 6B), the RMSD reached approximately steady values after about 1 ns, which was mostly less than 1.5 Å. By contrast, in previous simulations, less stable *β*-sheet configurations readily disrupted within several nanoseconds [33],[37]. These simulations were performed at 330 K and the equilibration run was short, 50 ps. On the other hand, our simulations were at 300 K, and prior to production run we performed 2-ns MD in the GBSW implicit solvent, and then 1-ns equilibration in explicit water, to allow side chains in the bilayer to pack as much as possible. PBC provides additional stability by preventing exposure of reactive backbone hydrogen and oxygen [40]. Even so, we observed that non-native bilayer patterns have higher binding free energy than the native conformation.

**Figure 7:**
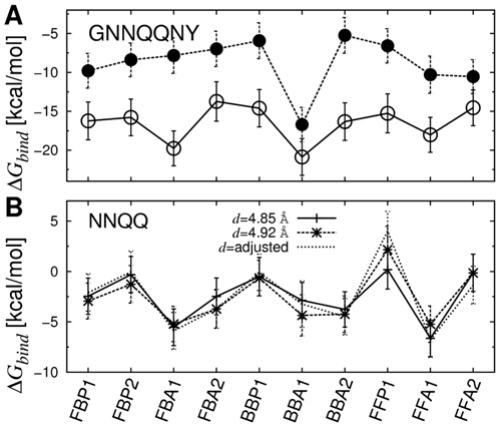
Free energy profiles of parallel *β*-sheets (GNNQQNY and NNQQ). (A) Open/solid circle: calculation based on MD with/without PBC in the filament axis. The exposed edge in the case without PBC elevates the overall energy level. (B) In the case when the inter-peptide distance d was adjusted, we used the CPT dynamics to maintain a constant pressure while the axial length of the simulation box fluctuated. Error bars in all graphs denote standard deviations. Values of individual energy terms are in Tables 2 and S2.

**Figure 8:**
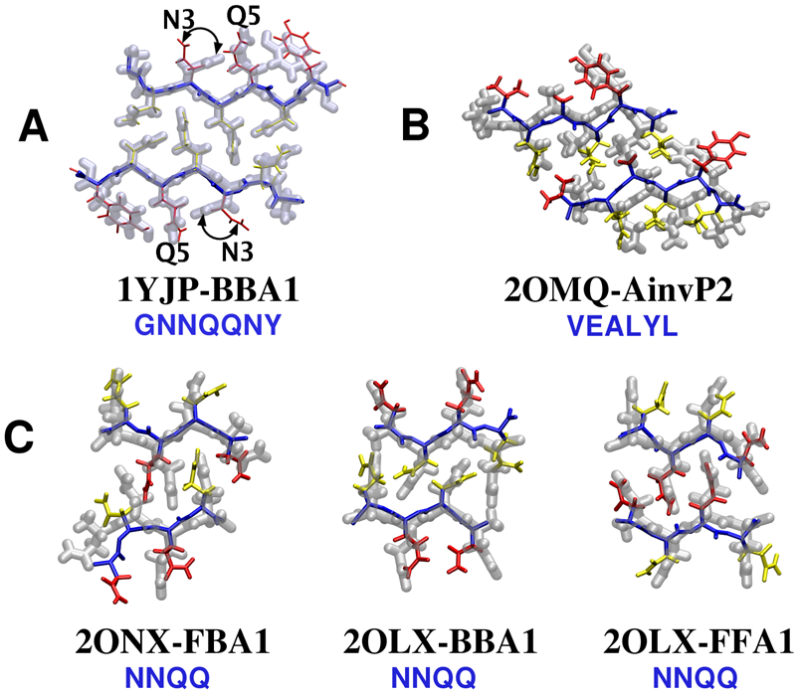
Comparison between PDB structures (transparent tube) and native-like candidates (wireframe). Snapshots at 2 ns of the production run were used to compare with x-ray crystallographic coordinates. (A) GNNQQNY, (B) VEALYL, and (C) NNQQ.

In the first set of simulations, we fixed the inter peptide distance (*d*; Table 1) over the course of simulation by imposing a PBC (see Methods). Among candidate patterns, BBA1 had the lowest 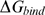, with the difference from the next lowest one being 1.11 kcal/mol (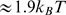; 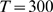 K. Free energy was measured on a per peptide basis) (Fig. 7A, open circles). BBA1 corresponded to the x-ray structure; although it was constructed from initially flat *β*-sheets (Fig. 2A, bottom), RMSD of heavy atoms from 1YJP was quite small, on average 1.18 Å. Hydrogen bonds between polar side chains at the dry interface (N2, Q4, and N6) were also observed, as in 1YJP. The only major difference was in the orientation of the N3 side chain (Fig. 8A, arrows). In 1YJP, it points to Q5 to form a side chain hydrogen bond. However, N3 and Q5 are exposed to water and would remain individually solvated, so the N3-Q5 hydrogen bond is likely a crystallization artifact in 1YJP. In fact, even when we imposed the N3-Q5 side chain hydrogen bond in the starting structure, it broke and the side chain of N3 rotated to the other way during the simulation.

To check if the selection of BBA1 is robust, we performed additional tests. The profile of 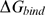 averaged locally over 1-ns intervals showed a consistent trend (Fig. S1A). Although less stable or non-native patterns had slightly more variation in locally averaged 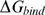 over time, most patterns maintained their structural states, and the selection of BBA1 is clear from the the beginning of the production run (cf., Fig. S4). This is likely due to the long initial preparatory simulations and the application of PBC mentioned above.

We also repeated simulations for the most and a moderately stable patterns (BBA1 and FFA1) with system sizes of 20 peptides instead of 12, which resulted in no major difference (Table 2). Among other peptides that we tested, although the relative stability between a few patterns could not be distinguished, using 12 peptides gave generally satisfactory result. Note that the computational cost sharply increases with the system size, since there are several patterns to test for a given peptide, and NMA also has strong size dependence.

**Table 2:** Decomposition of 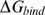 of GNNQQNY bilayers.

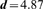 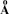	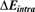	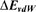	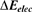	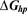	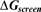	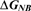	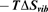	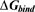
FBP1	−2.53	−29.11	107.09	−12.34	−102.18	−36.54	−1.08	−16.72
FBP2	−1.70	−28.98	128.95	−12.54	−124.15	−36.72	−1.23	−16.27
FBA1	−1.22	−30.18	−33.66	−12.78	34.75	−41.87	−0.55	−20.25
FBA2	−2.57	−25.03	9.93	−10.87	−6.45	−32.42	−2.68	−14.22
BBP	−1.19	−30.37	109.47	−12.64	−104.19	−37.73	0.42	−15.09
**BBA1**	−3.24	−30.87	47.51	−13.36	−44.84	−41.56	0.05	−21.36
BBA1	(−3.41)	(−30.65)	(44.10)	(−13.41)	(−42.50)	(−42.46)	N/A	N/A
BBA2	−2.06	−29.50	25.74	−12.55	−19.23	−35.54	−0.88	−16.74
FFP	−2.12	−28.91	108.02	−12.24	−102.25	−35.38	−1.09	−15.74
FFA1	−1.46	−29.23	0.60	−12.82	1.20	−40.25	−0.33	−18.38
FFA1	(−1.12)	(−29.48)	(13.39)	(−13.19)	(−13.12)	(−42.40)	N/A	N/A
FFA2	−0.20	−26.76	12.29	−11.68	−10.12	−36.27	−2.00	−15.03

The most stable pattern (BBA1) is marked in bold, which also corresponds to the x-ray structure. The energy unit is kcal/(mol peptide) and temperature (*T*) is 300 K. 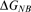 is the sum of the four terms on its left (Eq. 1). Translational and rotational free energies for a monomer were 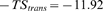 kcal/mol and 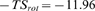 kcal/mol, which were subtracted from 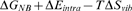 when calculating 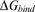. Numbers in parentheses are obtained from simulations with 20 peptides. Due to size limitations of the NMA facility in CHARMM, 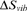 was not calculated in the 20-peptide case under PBC, but it is expected to have only a minor contribution. Although energy terms individually vary between simulations with 12 and 20 peptides, 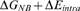 in BBA1 is consistently lower than that of FFA1 by 2.92 kcal/mol (12 peptides) and by 2.35 kcal/mol (20 peptides).

To test the effect of helical twist [36] as well as the exposed edge, we performed additional MD simulations of the *β*-sheet bilayer candidates without the PBC along the filament axis. All bilayers did not dissociate and developed curvature, although the small system size made it difficult to characterize their helical pitch. BBA1 was still the most stable (Fig. 7A, solid circles). Variation in energy due to changes in helical twist is less than that from different supramolecular packing patterns [46], so the flat bilayer structure under PBC can be used to distinguish the relative stability among candidate patterns. Also note that the free energy difference between BBA1 and the rest is larger without PBC (Fig. 7A), indicating that less stable structures suffer more from the edge effect.

### Polymorphism of NNQQ 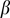-sheet bilayers

Unlike 1YJP that has one dominant energy minimum, if there are multiple minima with similar stability, molecular polymorphism may be possible. As a test, we applied the present approach to the peptide NNQQ, which has two x-ray structures differing in *β*-sheet packing patterns with distinct faces forming dehydrated interfaces and different inter-peptide distance, *d* [27]. Construction of *β*-sheet bilayers (Fig. 2B), MD simulation, and calculation of 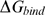 followed the same procedure as for GNNQQNY. Candidate patterns were tested under PBC in two different sets of simulations with inter-peptide distances within a *β*-sheet, 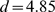 Å (2ONX) or 4.92 Å (2OLX) (Note that *d* does not change under the rigid PBC; see Methods).

When 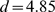 Å, FFA1 was the most stable pattern, although the native-like pattern was FBA1, with a small, 0.66 kcal/mol difference in 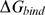. (Fig. 7B and Table S2). This can be explained in terms of the interaction between two *β*-sheet bilayers. In the case of 1YJP, a dehydrated steric zipper is formed only between the *β*-sheets in the BBA1 pattern, while there are crystal water molecules outside. However, in both 2OLX and 2ONX, there is no crystal water and both sides of the *β*-sheet bilayer form additional steric zipper interface with neighboring sheets. In the case of FBA1, it can repeat itself to build a laminated crystal. But when two FFA1 filaments stack, they must form a BB-pattern in between; BBP, BBA1, or BBA2 (Fig. S2). Our calculation shows that BBA2 is the most stable among BB-patterns. Therefore, rather than individually, the *average* 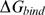 of FFA1 and BBA2 should be compared with that of FBA1, where the former average is 0.48 kcal/mol higher. Although this is in agreement with the selection of FBA1 in 2ONX, the energy difference is narrower than the thermal energy at 300 K (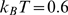 kcal/mol).

When 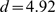 Å, the three most stable patterns were FFA1 (−5.23 kcal/mol), FBA1 (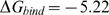 kcal/mol), and BBA1 (−4.35 kcal/mol). The native-like patterns are BBA1 and FBA1, whose average 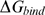 is only 0.44 kcal/mol (0.73 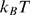) higher than that of FBA1. Such indeterminacy is presumably due to the symmetric sequence of NNQQ, which has the same side chains in the same order on both faces. Consistent with our result, an *ab initio* calculation indicated similar stability of the two crystal lattices [57].

Additional simulations support the above result. To allow the system to choose the inter-peptide distance *d* instead of fixing it by imposing a rigid PBC, we used a constant temperature and pressure (CPT) dynamics, where dimensions of the simulation box parallel and normal to the filament axis were controlled to keep the pressure at 1 atm, while PBC was still maintained. Averaged over the 2-ns production run, values of *d* were consistent with those from crystal structures: 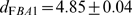 Å ( *cf.,* 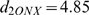 Å), 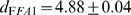 Å, and 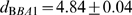 Å ( *cf.*, 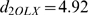 Å). The first and the second lowest 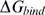 configurations were FFA1 and FBA1, respectively, consistent with results with a rigid PBC (Fig. 7B). FBA1 had 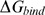 lower than the average between FFA1 and BBA1 by 0.88 kcal/mol, again comparable to 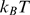. For the case where FF and BB patterns alternate (Fig. S2), we find that FFA1 is more stable than BBA1. Thus the FFA1 bilayer may form first, which subsequently stack to form the BB-interface.

As in the case of GNNQQNY, the most stable *β*-sheet bilayer patterns of NNQQ closely followed those of the respective x-ray structures. The RMSD of heavy atoms between the FBA1 structure at the end of the production run and 2ONX was 2.07 Å(rigid PBC) and 1.85 Å (CPT), and the RMSD between 2OLX and BBA1 and FFA1 was on average 1.89 Å (rigid PBC) and 2.0 Å (CPT) (Fig. 8C).

### Native anti-parallel filament patterns of VEALYL

**Figure 9:**
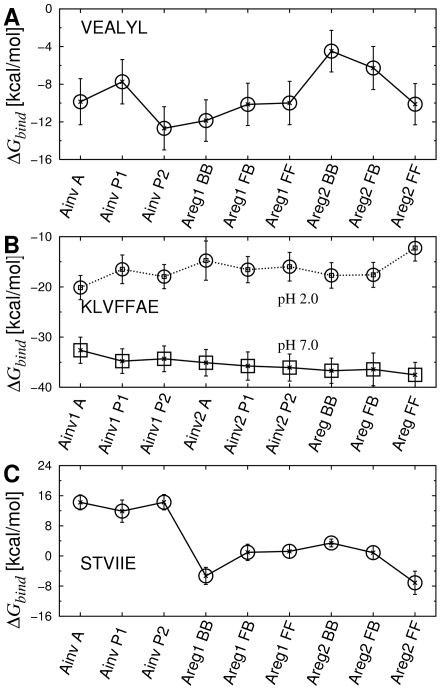
Free energy profile of anti-parallel *β*-sheets. (A) VEALYL, (B) KLVFFAE, and (C) STVIIE. Values of individual energy terms are in Tables S3–S5.

Our approach was effective in calculating 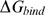 of anti-parallel *β*-sheet filaments as well. Out of nine candidate patterns considered (Fig. 4A), the most stable configuration for VEALYL was the native-like AinvP2, with 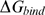 lower than the second lowest Areg1BB by 1.00 kcal/mol (Fig. 9A; Table S3). As explained in Discussion, the selection of an Ainv over an Areg pattern could also be driven by electrostatics at early stages of assembly, as the negatively charged E2 side chains are further apart in an Ainv sheet. Heavy atoms of the AinvP2 filament had an RMSD of 2.73 Å from 2OMQ (Fig. 8B). RMSD between other filament patterns and 2OMQ were significantly larger, *e.g.*, AinvP1 has 6.04 Å and Areg1BB has 9.10 Å. Furthermore, RMSD of the initially extended AinvP2 structure before the MD was on average 3.25 Å from 2OMQ, suggesting that AinvP2 structure indeed approached the x-ray structure after MD, sufficiently distinguishable from other patterns. As in GNNQQNY, calculation of 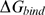 over 1-ns intervals confirmed that the free energy profile across different patterns established almost from the beginning of the production run (Figs. S1 and S5).

### Predicting unknown 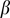-sheet bilayer structures of KLVFFAE and STVIIE

**Figure 10:**
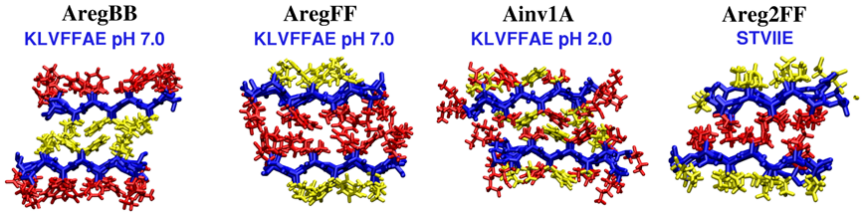
Most stable anti-parallel *β*-sheet bilayer configurations of A*β*_(16−22)_ (KLVFFAE) and STVIIE.

As we were able to identify the bilayer structures for peptides with known crystal structures, we applied our method to two peptides KLVFFAE and STVIIE, whose atomistic *β*-sheet bilayer structures are currently unknown. For KLVFFAE (A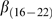), we tested pH 7.0 and 2.0, at which the peptide self-assembles into fibers and nanotubes, respectively [48]. At pH 7.0, our calculation indicated that AregFF is the most stable configuration, with 0.81 kcal/mol difference in 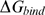 to the next lowest configuration AregBB (Fig. 9B, Fig. 10, and Table S4). Since the difference is marginal, as in the case of NNQQ (Fig. S2), AregFF and AregBB may stack to form a laminated fiber. Previously AregFB was suggested as the native *β*-sheet bilayer pattern [48], where simulations were performed for 0.8 ns and the *d*/2 axial shift between two layers (see Methods) was not implemented. Their main criterion for selecting the bilayer pattern was the inter-layer distance, to match the 9.9-Å x-ray fiber diffraction peak. If we use the average distance between 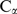 atoms in even- or odd-numbered residues as the inter-layer distance used in [48], we get, on average, 9.9 Å(AregBB), 10.9 Å (AregFF), and 9.5 Å (AregFB), where the last value is less than that from simulations in [48] possibly due to longer relaxation runs and the d/2 axial shift in our case. Although our result favoring the AregFF patterns differs from the AregFB pattern suggested in [48], at least the selection of an Areg pattern is consistent with existing solid state NMR data [22]. Further experiments would be necessary to clarify the structural selection and the atomic origin of the 9.9-Å peak.

However, when 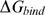 averaged over 1-ns intervals are followed, at pH 7.0, energies of Ainv2P1 and Ainv2P2 decreased significantly over time, almost to the lowest levels (Fig. S7). To further test if this is due to any finite size effect, we performed additional simulations on Ainv2P1, Ainv2P2, and AregFF using 10 peptides per *β*-sheet, where the production run lasted 4 ns. Based on 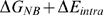, Ainv2P1 and Ainv2P2 became even more stable compared to AregFF (Table S4). As explained in Discussion, although the Ainv bilayer might in fact be more stable than the Areg pattern, the latter may be kinetically selected at the single sheet level.

At pH 2.0, by contrast, Ainv1A was the dominant free energy minimum (Fig. 9B), which nicely agrees with the result in [48]. In the case of STVIIE, candidate *β*-sheet bilayer configurations were similar to those of VEALYL (Figs. 3C and 4A). We found that the Areg2FF pattern was the lowest in 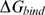 (Figs. 9C, 10, and Table S5). The selection was again clear, since the next most stable Areg1BB was 2.0 kcal/mol higher in 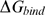.

## Discussion

The present results suggest that the binding free energy 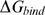 can be used to identify the actual cross-*β* bilayer structure that the peptide assembles into. We find that polymorphism is possible when there are similarly stable structures [18]. However, our results do not preclude the possibility that a structure can be selected kinetically during early stages of assembly over ones with 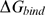 lower by more than 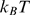: If the nucleus for the kinetically trapped structure is stable enough to persist and grow into filaments [28], any initial bias may result in its domination. As explained below, analysis of individual energy terms comprising 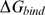 provides further insights into how different interactions might operate at various stages of structural evolution from early oligomers to mature fibrils.

### Driving forces for the structural selection

The binding free energy (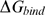) is composed of terms defined in Eq. 9, which can be grouped into non-bonded energy (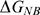), intramolecular (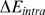), and entropic (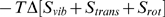) contributions. 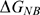 consists of 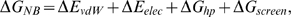
(1)where 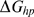 and 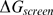 are respectively hydrophobic and electrostatic screening energies. Comparison among energy terms in 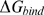 reveals that 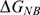 is dominant over 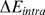 and entropic contributions (Table 2 for GNNQQNY, and Tables S2–S5 for other peptides). This suggests that the peptide does not become internally strained or relaxed (small 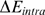), and it loses only small amount of vibrational entropy upon incorporating into the *β*-sheet bilayer: The bilayer structure is determined predominantly by non-bonded interactions.

Next we compared the four terms in Eq. 1 among candidate bilayer patterns. For GNNQQNY, 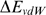 and 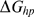 contribute greatly for the native-like BBA1 pattern compared to others, while there is no particular preference for BBA1 in 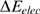 and 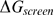 (Table 2). Absence of charged side chains in GNNQQNY accounts for the little role played by electrostatic interactions (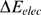). Unlike typical amyloidogenic peptides, GNNQQNY only has polar side chains, so that hydrophilic effect mediated by the surrounding shell of water molecules may play an important role in initially bringing these peptides into loosely formed aggregates, as in the assembly of collagen [58],[59]. However, since water molecules between the two *β*-sheets are eventually expelled, hydrophilic effect including 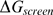 is not crucial for determining the side chain registry at the steric zipper interface. Therefore, although hydrophobic and van der Waals forces may not drive individual peptides in initial aggregation, they should be important in the final cross-*β* structure selection, where side chains within the bilayer pack by making direct contacts.

For VEALYL, all three terms except for 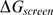 generally favor the native-like AinvP2 pattern (Table S3). An anti-parallel inverse *β*-sheet (Ainv) may have been favored since it has a single charged residue (E2) placed alternatively on the two faces of a *β*-sheet, which was also observed for KLVFFAE at pH 2.0. However, this is not a rigid rule, since STVIIE favored Areg2FF. In an Ainv-type *β*-sheet of STVIIE, rows of side chains are formed by T2–I5, and V3–I4. Due to the large size of the Ile side chain compared to those of T2 and V3, both faces of an Ainv *β*-sheet are uneven, which is disadvantageous for steric zipper formation. By contrast, the Areg2FF pattern has the row of V3 side chains from each layer at the core of a tightly formed steric zipper interface (see below). For NNQQ, there was no particular preference for native patterns in any of the four energy terms in 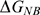 (Table S2). Since the two faces of a parallel NNQQ *β*-sheet have identical side chains, they are nearly equally likely to form steric zipper interfaces, and native polymorphic patterns (BBA1+FFA1 and FBA1) are favored only through the sum of all energy terms.

### Energetics of the monomer 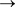 monolayer 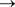 bilayer hierarchy

**Figure 11:**
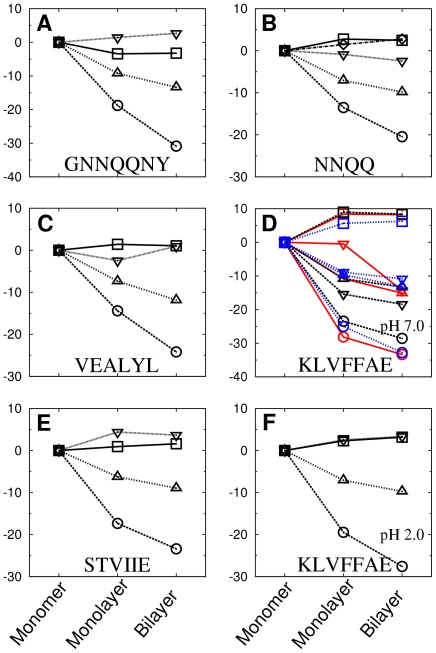
Free energy change accompanying the native bilayer formation. The y-axis is in units of kcal/mol. (A) BBA1 of GNNQQNY, (B) FFA1 (d = 4.92Å) of NNQQ, (C) AinvP2 of VEALYL, (D) AregFF (anti-parallel, black), Ainv2P2 (anti-parallel, blue), and FFA1 (parallel, red) of KLVFFAE at pH 7, (E) Areg2FF of STVIIE, and (F) Ainv1A of KLVFFAE at pH 2. Circle: Δ*E_vdW_*, triangle: Δ*G_hp_*, square: Δ*E_intra_*, and inverse-triangle: Δ*E_elec_* + Δ*G_screen_*.

The observation that van der Waals and hydrophobic interactions play a major role in selecting the bilayer pattern implies that interactions among side chains forming the dehydrated steric zipper, rather than among those exposed to water, are the major structural determinant. As an additional test, we analyzed free energy changes from a monomer to a single *β*-sheet, then to a *β*-sheet bilayer for the native-like pattern (Fig. 11). Although assembly from a monomer to a bilayer proceeds likely through multiple complex pathways, analyzing the free energy of a single *β*-sheet illustrates the role of steric zipper for stabilizing a bilayer. We evaluated the binding free energy of a *β*-sheet monolayer by ignoring one layer of *β*-sheet in double layer simulation trajectories. Since we use a continuum solvent model for free energy calculation, solvation effect is correctly taken into account even for the face that is originally buried in the bilayer. Similarly, in a previous study, the free energy of the monomer in a protein dimer was evaluated by ignoring the other monomer, yet the resultant free energy was comparable to that calculated from an isolated monomer simulation [51]. In contrast, MD simulation of a single Preg *β*-sheet of GNNQQNY (Fig. 3) showed strong tendency to twist and became unstable under PBC. While other single *β*-sheets might be more stable, we did not further investigate this since it was not necessary for our calculation and our major focus was on the *β*-sheet bilayer filament as the major building block of amyloid fibrils suggested by experiments [3],[27].

For all peptides tested, van der Waals energy (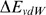) was reduced the most from a monomer to a single *β*-sheet and then to the native bilayer. Hydrophobic energy (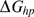) contributed the second (Fig. 11). On the other hand, 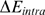 and 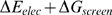 (the total electrostatic interaction) changed marginally, except for KLVFFAE at pH 7.0. Since the two charged side chains K1 and E7 lie on the same side of KLVFFAE, formation of an anti-parallel in-register *β*-sheet may be favored by electrostatic interactions, resulting in the AregFF pattern (Table S4). In the section ‘Possibility of hierarchical pattern selection in KLVFFAE,’ we discuss how the AregFF pattern might be kinetically preferred over other potentially more stable patterns such as Ainv.

### Importance of side chains forming the steric zipper interface

**Figure 12:**
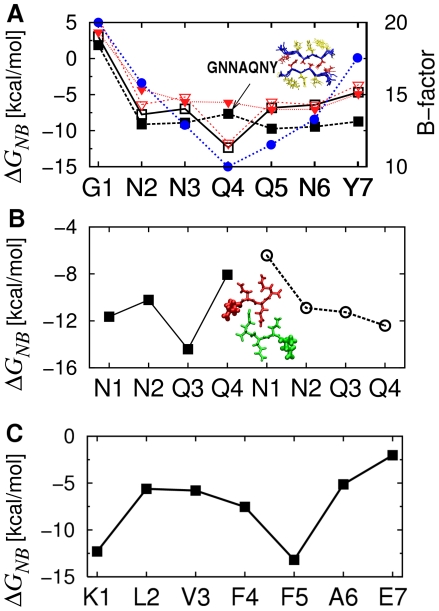
Per-residue contribution to ΔGNB. (A) BBA1 of GNNQQNY (open square and open inverted triangle) and GNNAQNY (solid square and solid inverted triangle), where data for black square and red inverted triangle are based on the monomer energy calculated from the standard procedure in Fig. 1, and by the REMD simulation, respectively. Blue solid circle: the average B-factor of each residue in the 1YJP structure. Compared to Q4, A4 has higher Δ*G_NB_* relative to other residues. The inset shows the cross section of the Q4A filament after the simulation, indicating a well-formed steric zipper interface. (B) FBA1 (d = 4.85Å) of NNQQ. Square (circle) represents each residue in the upper (lower) layer of *β*-sheet and N1 is marked in the picture to distinguish the direction of peptides. (C) AregFF of KLVFFAE.

Contribution by each residue to 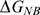 also supports that side chains at the steric zipper interface play a greater role compared to those exposed to water. For BBA1 of GNNQQNY, the residue-based profile of 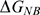 is consistent with its average B-factor in 1YJP (Fig. 12A). The greatest contribution is by Q4 located at the core of the steric zipper, followed by N2, revealing their stabilizing role. Odd-numbered residues facing water have comparatively higher 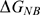. For G1, 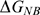 is the highest, thus it plays a minimal stabilizing role. This is consistent with the similarity between 1YJP and the structure without G1 (NNQQNY, PDB ID: 1YJO) [26]. Similar trends were observed for NNQQ and KLVFFAE, where side chains between the bilayer had greater contributions to 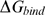 (Fig. 12B and C).

To gain further insight into the importance of side chain interactions in the bilayer interface, we tested the BBA1 bilayer formed by the Q4A mutant (GNNAQNY). When comparing 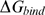 between patterns formed by different peptides, care should be taken since there is an uncertainty in the absolute magnitude of the free energy of monomers. To be clear, we performed two different types of monomer simulations (see Methods): The 1.6-ns monomer simulation in explicit-water at 300 K as used for most systems (Fig. 1), and replica-exchange molecular dynamics (REMD) in the GBSW implicit solvent, with a total simulation time of 1.6 *µ*s. In both cases, the peptides maintained mostly *α*-helical conformation (Methods; see also Fig. S9). Yet there were differences in the calculated energy of the monomer, which resulted in the BBA1 pattern of the Q4A mutant having 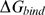 *lower* than that of GNNQQNY by 2.6 kcal/mol when the monomer simulation as in Fig. 1 was used, but *higher* by 3.91 kcal/mol when the REMD was used to calculate the monomer energy. As mentioned above and further explained in Methods, due to the uncertainty in calculating the absolute magnitude of the free energy of the monomer, it is difficult to conclude whether the Q4A mutant forms a more or a less stable BBA1 bilayer. However, compared to Q4 in GNNQQNY, A4 in GNNAQNY clearly has a decreased stabilizing contribution (increased energy per residue) relative to other residues in the peptide (Fig. 12A), which is consistent with the result in Ref. [37] on the destabilizing effect of the Q4A mutation. Yet the average inter-layer distance of the Q4A bilayer was 7.56 Å, which was narrower than that of the BBA1 pattern of GNNQQNY (9.25 Å). This is likely because the Ala side chain is smaller than the Gln side chain, making the Q4A mutant more advantageous to form a tighter steric zipper.

As an additional test, for GNNQQNY and GNNAQNY, we took the structures after the production run, kept 4 peptides in each layer, and performed explicit water simulations at 330 K with 1-ns equilibration followed by 4-ns production run without imposing a PBC. No disruption was observed in both filaments, contradicting the result in Ref. [37], where a Q4A mutant bilayer of the same size showed a strong destabilization at 330 K. This suggests that testing relative stability among candidate filaments using MD simulations at elevated temperatures does not guarantee that the most stable one survives the longest. Due to the finite (and usually small) system size, thermal disruption is a stochastic event, so even the most stable pattern may break earlier than less stable ones, which would be especially the case when the difference in stability is small. Conversely, as widely observed in the present study, less stable filaments may not break within the finite simulation time. For a more reliable test of stability, statistical average over a large number of simulations for a given bilayer pattern is necessary, which would be computationally very demanding. Our approach, on the other hand, uses one simulation trajectory for each pattern and provides contributions to the free energy by individual residues. Although it may not accurately predict whether a given point mutation will prevent fibril formation, it quantitatively shows how the mutated residue changes its contribution. Our result permits the possibility of the Q4A mutant assembling into a cross-*β* bilayer filament, whether or not the mutant filament is more or less stable compared to the original GNNQQNY bilayer. Further experiments are necessary to clarify the amyloidogenic propensity of Q4A.

Overall, our analysis highlights the importance of forming a tight steric zipper interface in selection and stabilization of the bilayer pattern. The favorable van der Waals interaction stems from the tight side-chain interaction within a *β*-sheet as well as between two *β*-sheet layers. Moreover, solvent exposed surface area of each *β*-sheet is reduced by the formation of the steric-zipper interface [3]. It would be difficult for *β*-sheets with dissimilar side chains to form a tight steric zipper, which requires shape complementarity between two interfaces. This is consistent with the fact that, although amyloid fibrils can form over a wide range of amino acid sequences [60], each fibril is composed of peptides with the same or similar sequence [61].

### Choice between parallel vs. anti-parallel 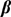-sheets

In a steric zipper, the row of side chains on a sheet along the filament fills the groove formed between two rows of side chains on the opposing sheet. Such a packing would be easier if side chains forming the row are identical or similar in shape, since the groove will then present a smooth interface. From the point of view of forming a steric zipper, a parallel *β*-sheet would thus be advantageous, which also has better side chain contacts within the row of side chains compared to an anti-parallel *β*-sheet, as suggested previously [62]. In contrast, as seen in VEALYL, KLVFFAE, and STVIIE, presence of charged side chains favors anti-parallel *β*-sheet due to electrostatic interactions. Indeed, among 15 crystal structures of cross-*β* spines published in [26],[27],[63], 11 are parallel *β*-sheets, none of which has charged residues. Among 4 anti-parallel *β*-sheet structures, LYQLEN and VEALYL have charged residues. The remaining 2 anti-parallel *β*-sheet structures are polymorphic forms of the peptide MVGGVV. Although MVGGVV has no charged residue, it does not have the disadvantage in forming a steric zipper between anti-parallel *β*-sheets mentioned above: The identical side chains of V2 and V5 can stack sideways in an anti-parallel *β*-sheet, and two consecutive glycines provide enough room for side chain arrangement, which may also be relevant to its polymorphism as well.

### Possibility of hierarchical pattern selection in KLVFFAE

As observed in Results (Table S4), KLVFFAE had two classes of lowest free energy *β*-sheet bilayer configurations, which were formed respectively by Ainv2 and Areg monolayer patterns (Fig. 3D). Although Ainv2 patterns could be comparably stable at the bilayer level, as a monolayer, 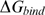 of Areg is 1.84 kcal/mol (6-mer per layer) or 0.78 kcal/mol (10-mer per layer) *lower* than that of Ainv2 ( *cf.*, Fig. 11D). This is presumably due to the favorable electrostatic interactions between charged residues (K1 and E7) in the Areg pattern, which agrees with previous experimental findings [22],[48]. This suggests that specific *β*-sheet bilayer pattern may be hierarchically determined from the most favored monolayer to the bilayer pattern.

As an additional test, we calculated 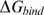 of the parallel FFA1 pattern ( *cf.*, Fig. 2A) formed by KLVFFAE at pH 7. It can be clearly seen that side chains at the interface pack better compared to anti-parallel *β*-sheets (Fig. 10 vs. Fig. S3). Surprisingly, the calculated 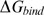 of FFA1 was −39.84 kcal/mol, which is 2.33 kcal/mol *lower* than that of the anti-parallel configuration, AregFF. However, when the comparison is made between *β*-sheet monolayers, 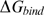 of the FFA1 pattern is 6.77 kcal/mol higher than that of AregFF, mainly due to unfavorable electrostatic interactions among like charges in the parallel *β*-sheet (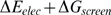; Fig. 11D). But when an FFA1 bilayer is formed, K1 and E7 from opposing layers form salt bridges, resulting in reduced electrostatic repulsion. Therefore, based on the free energy decomposition of different *β*-sheet patterns, it can be seen that, although a parallel or Ainv-type *β*-sheets are preferred at the bilayer level for KLVFFAE at pH 7.0 due to better side chain packing, there may be a kinetic barrier originating from strong electrostatic repulsion at the single sheet level, resulting in the selection of the Areg *β*-sheet to form a bilayer. However, the difference in 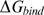 between the Ainv2 and Areg monolayers is marginal, especially in simulations with 20 peptides. Thus one cannot exclude the possibility of molecular polymorphism in KLVFFAE at pH 7.0.

It is conceivable that a mutant peptide KLVFFAQ at high pH and with multivalent ions may assemble into a parallel *β*-sheet filament. Since the only charged Lys residue of the mutant becomes neutralized and screened, the initial electrostatic drive for an anti-parallel *β*-sheet could be suppressed. However, the strong hydrophobic interactions by other residues may cause the system to collapse into amorphous aggregates. In such cases, reducing the solvent polarity, *e.g.*, by adding acetonitrile, could assist with *β*-sheet formation. In any case, once a *β*-sheet type is determined, the bilayer pattern can be predicted with a reasonable accuracy by comparing their 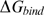. For longer peptides, side chain packing would dominate over electrostatic interactions, unless it has a proportionately large number of charged residues. Available solid-state NMR structures of amyloid fibrils composed of 40- or 42-residue long A *β* peptides are indeed parallel [23], [64]–[66].

To further test the possibility that the *β*-sheet type is selected at the monolayer level, we calculated 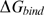 of other anti-parallel *β*-sheets, KLVFFAE at pH 2.0, VEALYL and STVIIE. For KLVFFAE at pH 2.0, the most stable monolayer was Ainv1, with 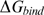 lower than the Ainv2 (Areg) pattern by 2.89 (3.61) kcal/mol. This is consistent with Ainv1A being the most stable bilayer pattern at pH 2.0 (Table S4). Similarly, the Areg2 monolayer of STVIIE forming the most stable Areg2FF bilayer had 1.24 kcal/mol lower in 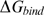 than Areg1 that forms the next most stable Areg1BB bilayer (Table S5). On the other hand, the Ainv monolayer of VEALYL (forming the most stable bilayer; Table S3) had 1.59 kcal/mol *higher* in 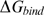 than Areg. Therefore, although generally the most stable *β*-sheet monolayer may be used to form the native-like bilayer, this is not universally applicable. As previously found [28], structural evolution of oligomers is affected by both kinetic and energetic factors depending on the conformational relaxation time as well as the peptide concentration.

### Role of backbone hydrogen bonds

It has long been suggested that backbone hydrogen bonds are major interactions in forming the amyloid cross-*β* structure [1]. In the CHARMM force field, the hydrogen bond energy is accounted for by the sum of electrostatic and van der Waals interactions between partially charged hydrogen bond donor and acceptor atoms. We decomposed 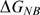 to calculate the hydrogen bond energy of each backbone H--O pair, which is on average −1.98 kcal/mol for the BBA1 pattern of GNNQQNY and −1.28 kcal/mol for the AinvP2 pattern of VEALYL. Since there are 5–6 backbone hydrogen bonds per peptide in the native GNNQQNY and VEALYL configurations (Fig. 3A), they contribute 24% (18%) of 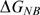 of GNNQQNY (VEALYL), which is indeed a significant fraction. Maximization of the number of backbone hydrogen bonds thus mainly favors in-register *β*-sheets over out-of-register ones. However, since we compare bilayers formed by in-register *β*-sheets that contain mostly the same number of backbone hydrogen bonds, hydrogen bonds cannot be a major determinant for the selection of a specific bilayer pattern.

### Conclusion

Our present analysis implies subtle roles played by kinetics and energetics in amyloid assembly. Kinetic trapping would be more relevant at early stages of assembly where basic features such as the *β*-sheet type (parallel vs. anti-parallel, or Ainv vs. Areg) are determined [28]. Once the *β*-sheet grows beyond the size of critical nucleus, it would be very difficult to change in any major way, such as adjusting the peptide registry within the sheet, or switching between parallel and anti-parallel types. In contrast to changes requiring major backbone hydrogen bond rearrangement, bilayer type selection would occur at a later stage, and more subject to an energetic control, because it involves shape complementarity between two faces that usually does not require any specific bond formation. Lack of specific bonds would allow conformational search and an optimal steric zipper packing would be achieved between two small *β*-sheets. Once such a fibril grows to a larger size, it will serve as a template for further growth, and structural rearrangement at the molecular level is unlikely, as experiments suggest [18]–[20]. Such a scenario is also consistent with a recent simulation of the aggregation of the GNNQQNY peptide, where initially formed parallel *β*-sheet dimers are stabilized by subsequent formation of a steric zipper bilayer [67].

The successful use of the binding free energy per peptide 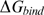 as the criterion for selecting the steric zipper pattern supports that the bilayer pattern is determined energetically. Molecular polymorphism would be possible if there are two or more most stable patterns with similar values of 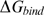, as was seen in NNQQ. However, our analysis is valid only when a given peptide sequence forms a *β*-sheet bilayer, and it does not address whether the peptide forms a cross-*β* filament or not in a certain buffer condition, for which different approaches have been developed [42]. Nor can our approach predict unusual cross-*β* structures such as with a bend (MVGGVV, PDB ID: 2OKZ) [27] or a turn (NNFGAIL, PDB ID: 3DGJ) [63]. Nevertheless, the ability to calculate the binding free energy is a significant advance since detailed analysis of the contribution by different energy terms provides quantitative explanation for the selection of a particular steric zipper pattern. Our approach would also be useful for identifying the most probable structure among multiple solid-state NMR structures [23], or for quantifying residue-specific contributions that may be therapeutically targeted for disruption of self-assembly.

## Methods

### Construction of peptides

Each peptide was modeled according to the experimental condition where its atomic coordinates were determined: GNNQQNY, NNQQ, VEALYL, and STVIIE had no capping moiety at both termini [27]. For KLVFFAE, the N- and C-termini were respectively acetylated and amidated [4]. Protonation status of titratable groups was determined based on pH of the corresponding experiments (Table 1).

### Construction of 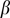-sheets

The type of a *β*-sheet and the inter-peptide distance *d* within a sheet were selected as summarized in Table 1 and Fig. 3. Two *β*-sheets were put together to form a bilayer filament pattern, with an initial inter-layer distance of 10 Å. One layer was then shifted axially by *d*/2 to maximize the interdigitation of side chains between the bilayer. Such a shift is present in various x-ray structures [26],[27]. Even when we started the simulation without the axial shift, it appeared spontaneously after the heating period in the implicit solvent environment, regardless of the boundary condition imposed on the filament axis.

### Structural relaxation in implicit solvent

For all simulations we used CHARMM version 31 [68] with the param22 all-atom force field. We performed preparatory simulations in the GBSW continuum solvent environment incorporated in CHARMM [52], to find proper side chain orientation. Lack of viscosity in GBSW aided rapid relaxation of side chains. Initially the system (either a monomer or one of the bilayer patterns) was relaxed through 3000 steps of energy minimization using the adopted basis Newton-Raphson (ABNR) algorithm. The system was heated from 0 K to 300 K for 60 ps, equilibrated at 300 K for 1.0 ns, followed by a 1.0 ns production run. The cutoff distance for the non-bonded interaction was 24 Å for the GBSW simulation. The final snapshot of each candidate was used as the initial structure for the explicit solvent simulation. We imposed a PBC to the filament axis by choosing the dimension of the simulation box parallel to the filament axis as *Nd*, where *N* is the number of peptides per layer.

### Explicit water molecular dynamics simulation

The final structure from the 2.06 ns implicit solvent simulation was put in an orthorhombic box containing TIP3 water molecules pre-equilibrated at 1 atm, 300 K. Water molecules were deleted whose oxygen atoms were within 2.9 Å from heavy atoms of the bilayer. The distance of 2.9 Å was chosen to ensure no water molecule was left within the bilayer after deletion, whereas the density of water was maintained by the constant pressure MD. The dimension of the solvation box was chosen large enough to prevent the interaction between the filament and its images except when a PBC was applied in the axial direction. In this case, the length of the box was the same as that of the filament. The transverse size of the box ranged between 50–66 Å, depending on the bilayer pattern tested. After putting water molecules, the system was energy minimized for 2000 steps using the ABNR method. Each configuration was heated for 100 ps then equilibrated for 1.0 ns. During equilibration, velocities were rescaled when temperature deviated from 300 K by more than ±5 K. A 2.0 to 6.0-ns production run followed (Table 1). When PBC was applied, the axial length of the filament (i.e. 29.2 Å for a system composed of 12 GNNQQNY peptides) was kept fixed while the transverse area of the simulation box fluctuated to maintain the constant pressure of 1 atm. In some cases, simulations were performed using the CPT dynamics where the axial length was adjusted (Table 1). Coordinates were saved every 0.5 ps during the production run. The cutoff distance for the non-bonded interaction was 12 Å for the explicit water simulation. We applied a similar procedure for a single peptide, which is required for the calculation of 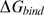.

### Calculation of the binding free energy (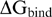)

We consider four states of a peptide: as a monomer or within a bilayer, either in vacuum or in solution: 
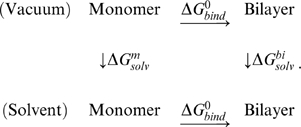
(2)

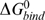
 is the free energy difference of the peptide in vacuum as an isolated monomer *vs.* in a bilayer; 
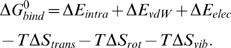
(3)

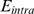
 includes covalently bonded energy terms associated with bond stretching, bond angle, and proper/improper dihedral angles. 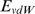 and 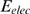 are van der Waals and electrostatic energies in vacuum. 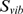, 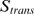 and 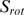 are vibrational, translational and rotational entropy contributions [56]: 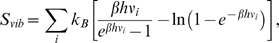
(4)

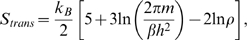
(5)

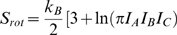
(6)

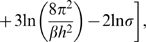
where 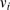 is the *i*-th normal mode frequency, *m* is the mass of a single peptide, 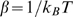, *h* is Planck constant, *ρ* is the number density (in units of M), *σ* is the symmetry factor of the molecule, and 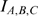 are three rotational moments of inertia. For the peptide within a bilayer, 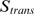 and 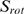 were set to zero. For convenience, *ρ* was set to 1 M. Although this is higher than typical experimental value, ∼1 *µ*M, a different choice only shifts 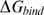 overall by a constant factor without affecting conclusions of the present work. *σ* was unity because the peptide is an asymmetric molecule.


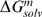
 is the solvation free energy of a monomer; 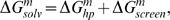
(7)where 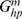 is the non-polar ‘hydrophobic’ energy, proportional to the solvent accessible surface area. 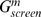 is the polar solvation free energy approximated by the generalized Born solvation model [52]. The GBSW facility in CHARMM was used to calculate these terms, which is known to reproduce the results calculated from the Poisson-Boltzmann equation approach within 2% errors [52].

Similarly, 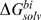 is the solvation free energy of the bilayer state; 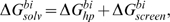
(8)which was again calculated using GBSW.

In the above, energy terms calculated for a bilayer were divided by the number of peptides in the bilayer. Finally, 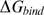, the Gibbs free energy of bilayer formation (per peptide), can be calculated as, considering Eq. 2, 
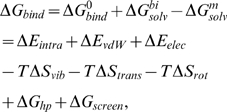
(9)where 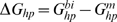 and 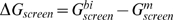.

After simulation, water molecules were deleted and energy terms except for entropic contributions were calculated for each frame, and averaged over each 1 ns period. To calculate 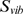, we took 10 snapshots per each 1 ns period, each of which was energy minimized and normal modes were calculated using the distance dependent dielectric constant (KLVFFAE and STVIIE) or in the GBSW solvation environment. The choice of solvation model may shift 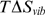 at 300 K by ±2 kcal/mol, but this does not affect our conclusion regarding relative stability among different bilayer patterns in a given solvent model. Eq. 4 was used to calculate the vibrational entropy, which was averaged over the snapshots to estimate 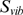.

We estimated the standard deviation of the calculated 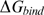 as follows. First 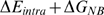 and 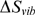 (Eq. 1) were averaged and respective variances, 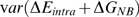 and 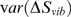, were calculated over the production run. For the monomer, we did not consider its energy fluctuation since the energy of monomer only affects the overall magnitude of 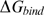 (see below). Since the variance of the sum of two independent random variables is the sum of individual variances [69], we get 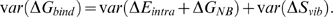
(10)


### Replica exchange molecular dynamics (REMD) simulation

In simulations, a monomer is more prone to conformational fluctuation than *β*-sheet bilayer filaments. Thus one should be careful in interpreting the magnitude of 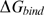. The fluctuation in the free energy of the monomer can induce an overall shift in the 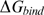 profile. Thus, although our approach is effective in comparing relative stability among bilayer patterns for a given peptide, it would be difficult to use the calculated 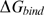 to address amyloidogenecity of a peptide, or to compare relative stability between bilayer patterns composed of different peptides.

For additional comparison between the stability of GNNQQNY and GNNAQNY bilayers, we performed REMD [70] for the corresponding monomers. We prepared 16 replicas of each monomer, with simulation temperatures spanning from 275 K to 600 K. The GBSW continuum solvation model was used. Temperature swap trials were attempted every 20 ps according to the Metropolis criterion and lasted for 100 ns, with a total simulation time of 1.6 *µ*s. During this period, each replica visited the lowest (highest) temperature at least more than 22 (69) times. 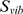 was calculated by energy minimizing the 5000 REMD structures at 300 K and performing NMA on each. Compared to the all-atom explicit water simulation of a monomer, 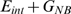 of GNNQQNY increased only by +0.40 kcal/mol, while for GNNAQNY, it decreased by −7.65 kcal/mol. Thus when the monomer energy based on the REMD simulation is subtracted (Eq. 9), 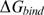 increases more for GNNAQNY than for GNNQQNY, which is opposite to the case when monomer energy from the constant-temperature simulation (Fig. 1) was used.

The DSSP algorithm [71] allowed detailed characterization of each monomer conformation at 300 K. The most abundant conformation of GNNQQNY was *α*-helix, with an occurrence probability of 58%. Hydrogen-bonded turn and *π*--helix appeared 13% and 11%, respectively (Fig. S9). However the above secondary structures possess very similar conformation, as the inset of Fig. S9 shows. This agrees with the corresponding constant temperature MD simulation, where *α*-helix was the dominant conformation. Similarly, GNNAQNY had *α*-helix (51%), *π*--helix (14%), and hydrogen bonded turn (11%).

## Supporting Information

Figures S1 Δ*G_bind_* profiles over successive 1-ns intervals. Black solid line indicates Δ*G_bind_* averaged over 4-ns production period. Although there are slight changes in Δ*G_bind_* over time, the overall profile is established from the beginning of the simulation. See Fig. S4-S8 for the time variation of locally averaged free energies.
(9.00 MB TIF)

Figures S2 Polymorphic crystal structures of NNQQ. Odd (even) numbered residues are colored in red (yellow).
(0.33 MB EPS)

Figures S3 Conformation of a parallel KLVFFAE β-sheet bilayer at pH 7.0 (FFA1).
(0.52 MB EPS)

Figures S4 Profile of Δ*G_bind_* versus time in GNNQQNY β-sheet bilayers. Each symbol represents the average over 1-ns interval.
(1.94 MB TIF)

Figures S5 Profile of Δ*G_bind_* versus time in VEALYL β-sheet bilayers. Each symbol represents the average over 1-ns interval.
(2.81 MB TIF)

Figures S6 Profile of Δ*G_bind_* versus time in NNQQ β-sheet bilayers. Each symbol represents the average over 1-ns interval.
(1.24 MB TIF)

Figures S7 Profile of Δ*G_bind_* versus time in KLVFFAE β-sheet bilayers. Each symbol represents the average over 1-ns interval.
(1.30 MB TIF)

Figures S8 Profile of Δ*G_bind_* versus time in STVIIE β-sheet bilayers. Each symbol represents the average over 1-ns interval.
(1.96 MB TIF)

Figures S9 Secondary structure distribution of GNNQQNY and GNNAQNY monomer. After completing REMD simulation, each snapshot at 300 K were analyzed using the DSSP algorithm. The *i*-th character in the name of each conformation represents the secondary structure of the corresponding amino acid; X: unstructured, B: β-bridge, S: bend, G: 3-helix, T: hydrogen bonded turn, H: α-helix, and I: π-helix.
(4.28 MB TIF)

Table S1 Correspondence between candidate patterns tested and eight steric-zipper classes proposed by Sawaya, et al. [27].
(0.01 MB PDF)

Table S2 Decomposition of ΔG*_bind_* of NNQQ bilayers. Native-like patterns are marked in bold. The energy unit is in kcal/(mol peptide)
(0.02 MB PDF)

Table S3 Decomposition of ΔG_bind_ of VEALYL bilayers. Native-like pattern is marked in bold.
(0.02 MB PDF)

Table S4 Decomposition of ΔG_bind_ of KLVFFAE bilayers. The most stable (possibly native-like) structures are marked in bold. Selected configurations in pH 7.0 were further simulated with larged system sizes (20 peptides) and corresponding energy values are in parentheses.
(0.02 MB PDF)

Table S5 Decomposition of ΔG_bind_ of STVIIE bilayers. The most stable (possibly native- like) structure is marked in bold.
(0.01 MB PDF)
