# Supplementary figures and images for: Thermodynamic Selection of Steric Zipper Patterns in the Amyloid Cross-β Spine
Source: PLoS Comput Biol. 2009 Sep 4;5(9):e1000492. doi: 10.1371/journal.pcbi.1000492 (PMC2723932; doi:10.1371/journal.pcbi.1000492)

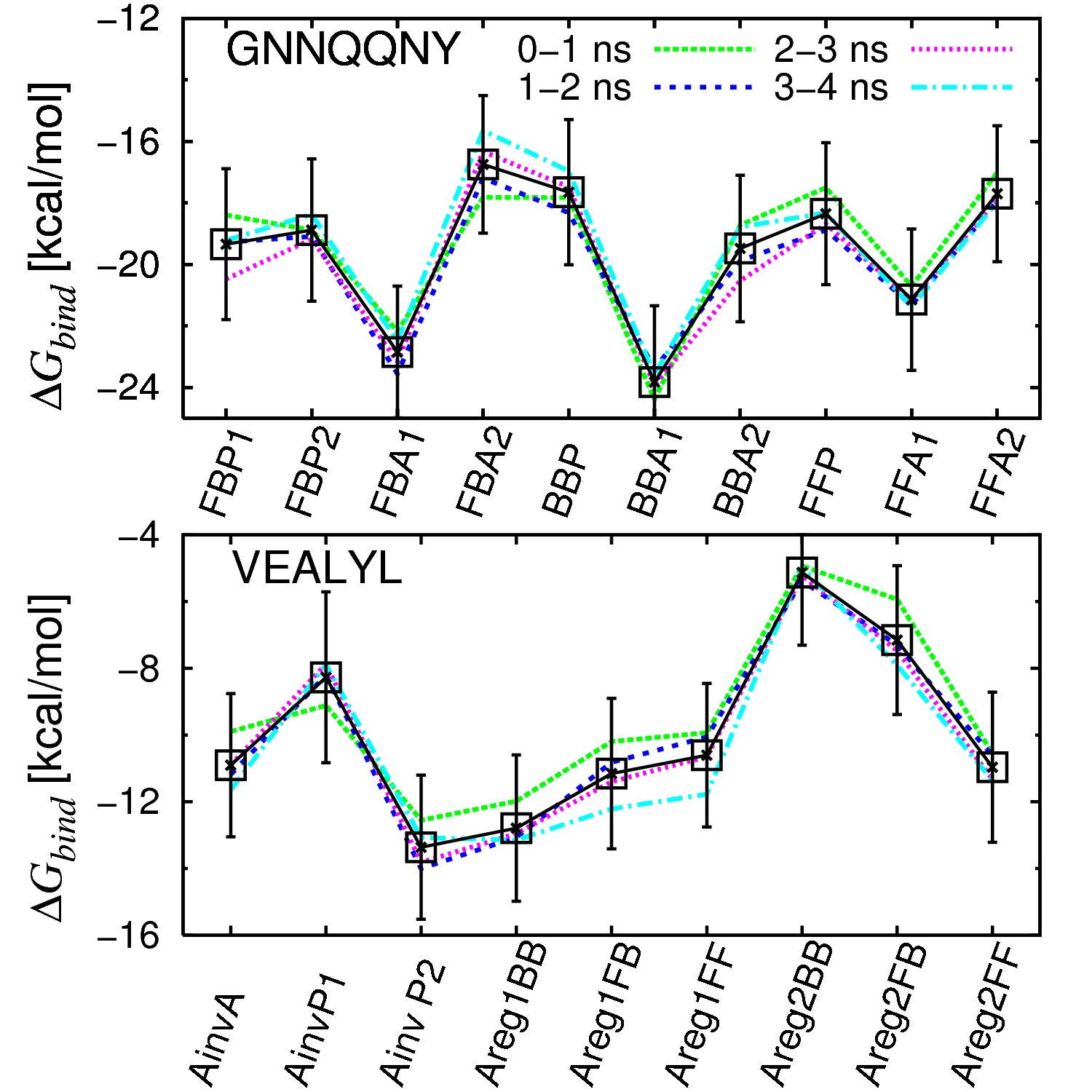

Supplement: Figures S1 — ΔGbind profiles over successive 1-ns intervals. Black solid line indicates ΔGbind averaged over 4-ns production period. Although there are slight changes in ΔGbind over time, the overall profile is established from the beginning of the simulation. See Fig. S4-S8 for the time variation of locally averaged free energies. (9.00 MB TIF) [file pcbi.1000492.s001.tif]

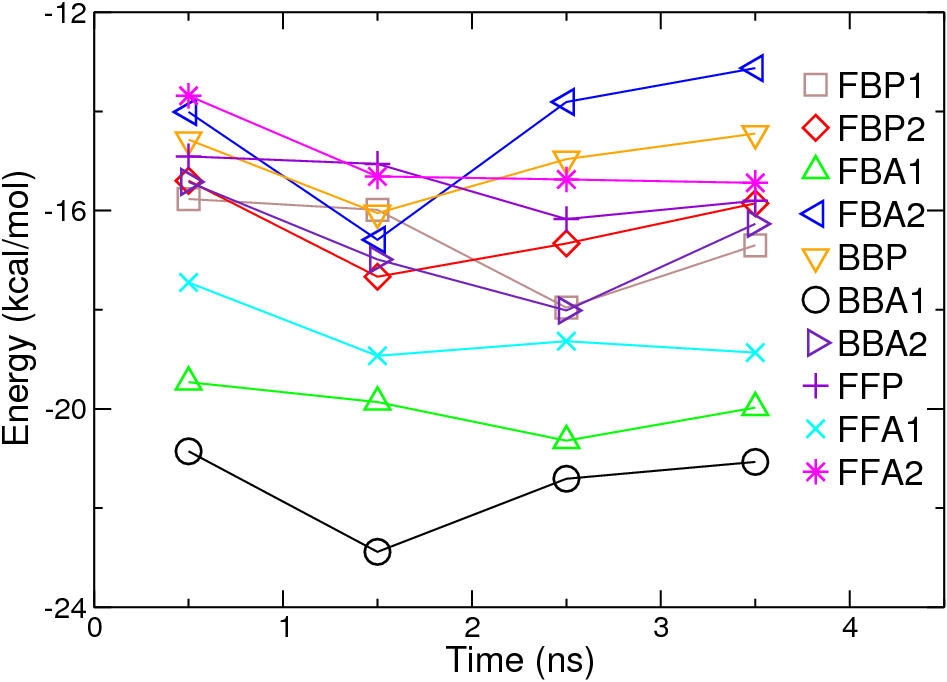

Supplement: Figures S4 — Profile of ΔGbind versus time in GNNQQNY β-sheet bilayers. Each symbol represents the average over 1-ns interval. (1.94 MB TIF) [file pcbi.1000492.s004.tif]

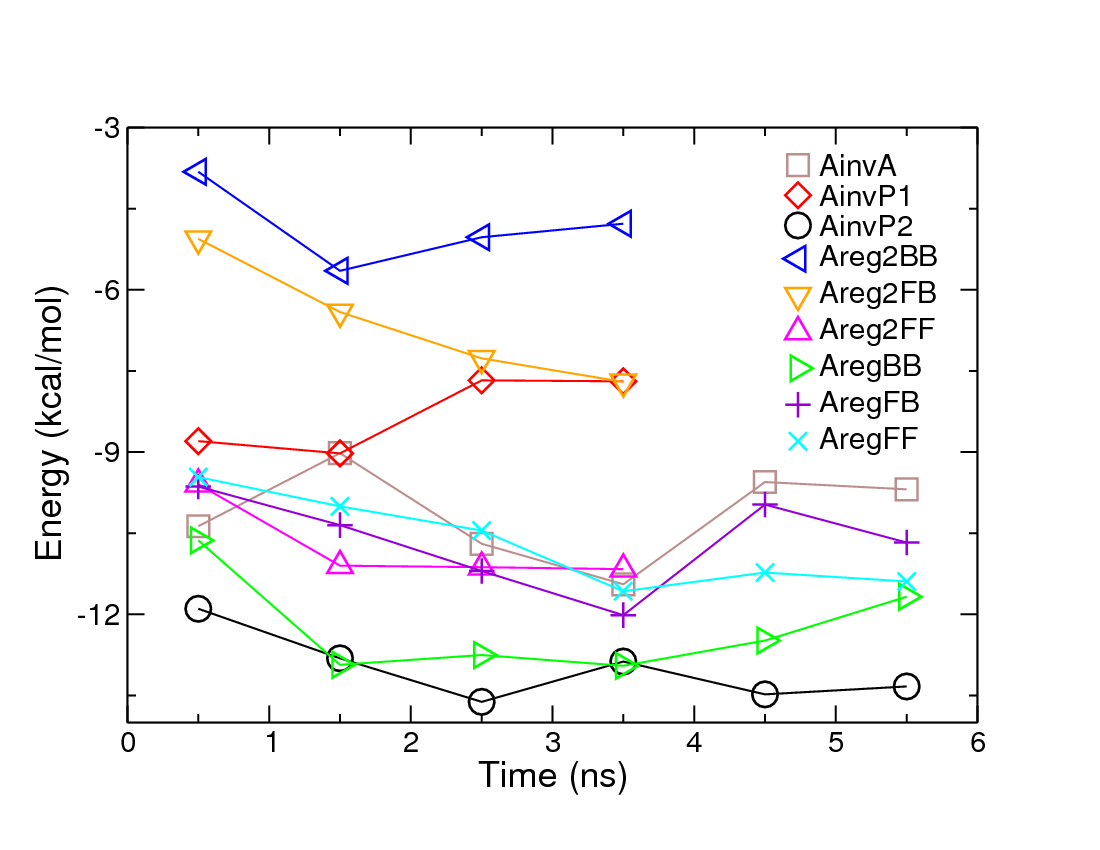

Supplement: Figures S5 — Profile of ΔGbind versus time in VEALYL β-sheet bilayers. Each symbol represents the average over 1-ns interval. (2.81 MB TIF) [file pcbi.1000492.s005.tif]

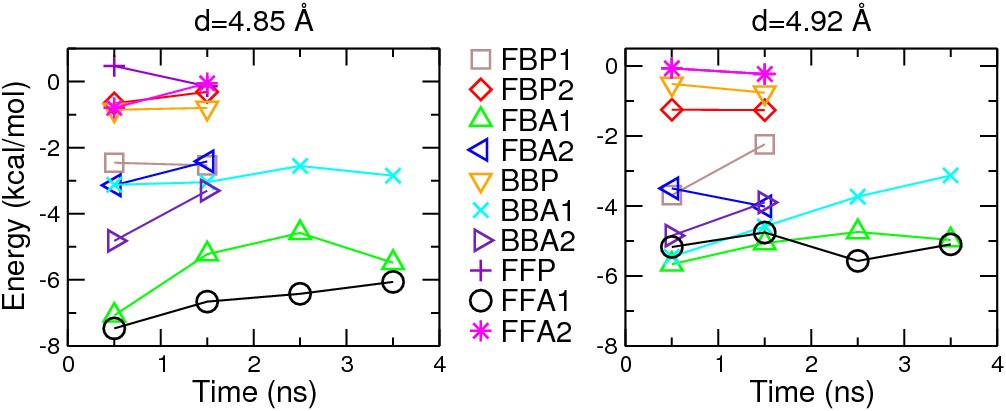

Supplement: Figures S6 — Profile of ΔGbind versus time in NNQQ β-sheet bilayers. Each symbol represents the average over 1-ns interval. (1.24 MB TIF) [file pcbi.1000492.s006.tif]

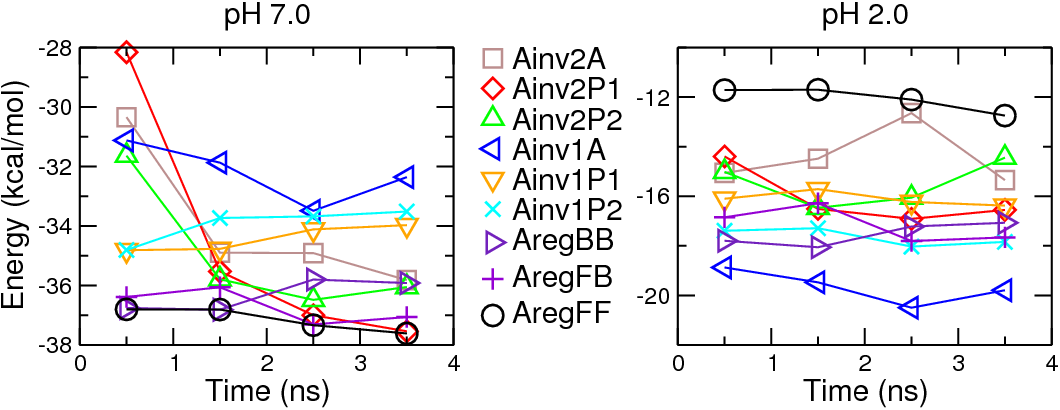

Supplement: Figures S7 — Profile of ΔGbind versus time in KLVFFAE β-sheet bilayers. Each symbol represents the average over 1-ns interval. (1.30 MB TIF) [file pcbi.1000492.s007.tif]

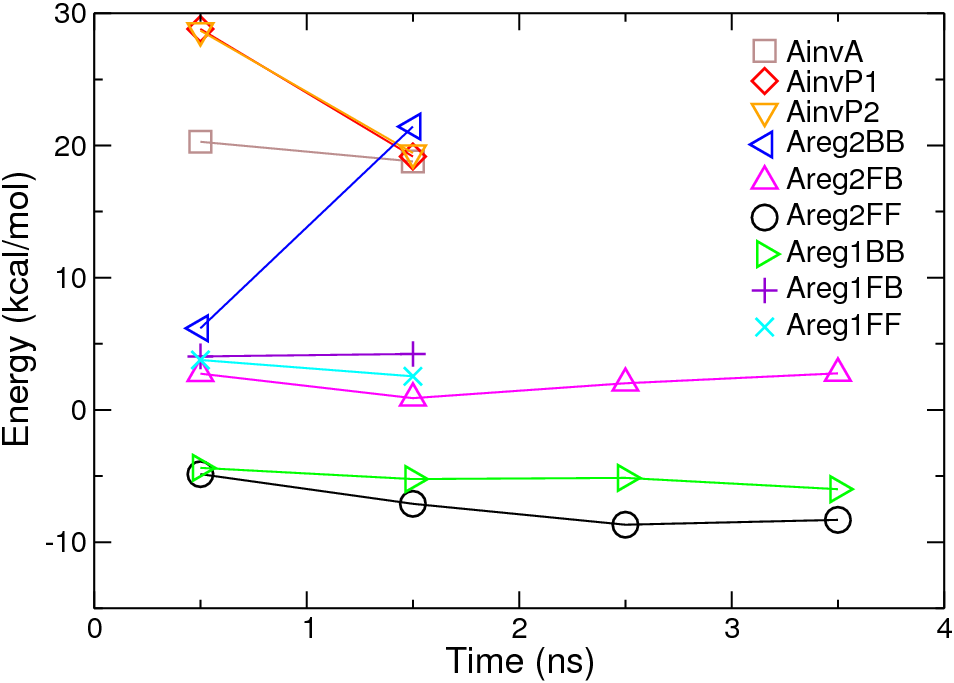

Supplement: Figures S8 — Profile of ΔGbind versus time in STVIIE β-sheet bilayers. Each symbol represents the average over 1-ns interval. (1.96 MB TIF) [file pcbi.1000492.s008.tif]

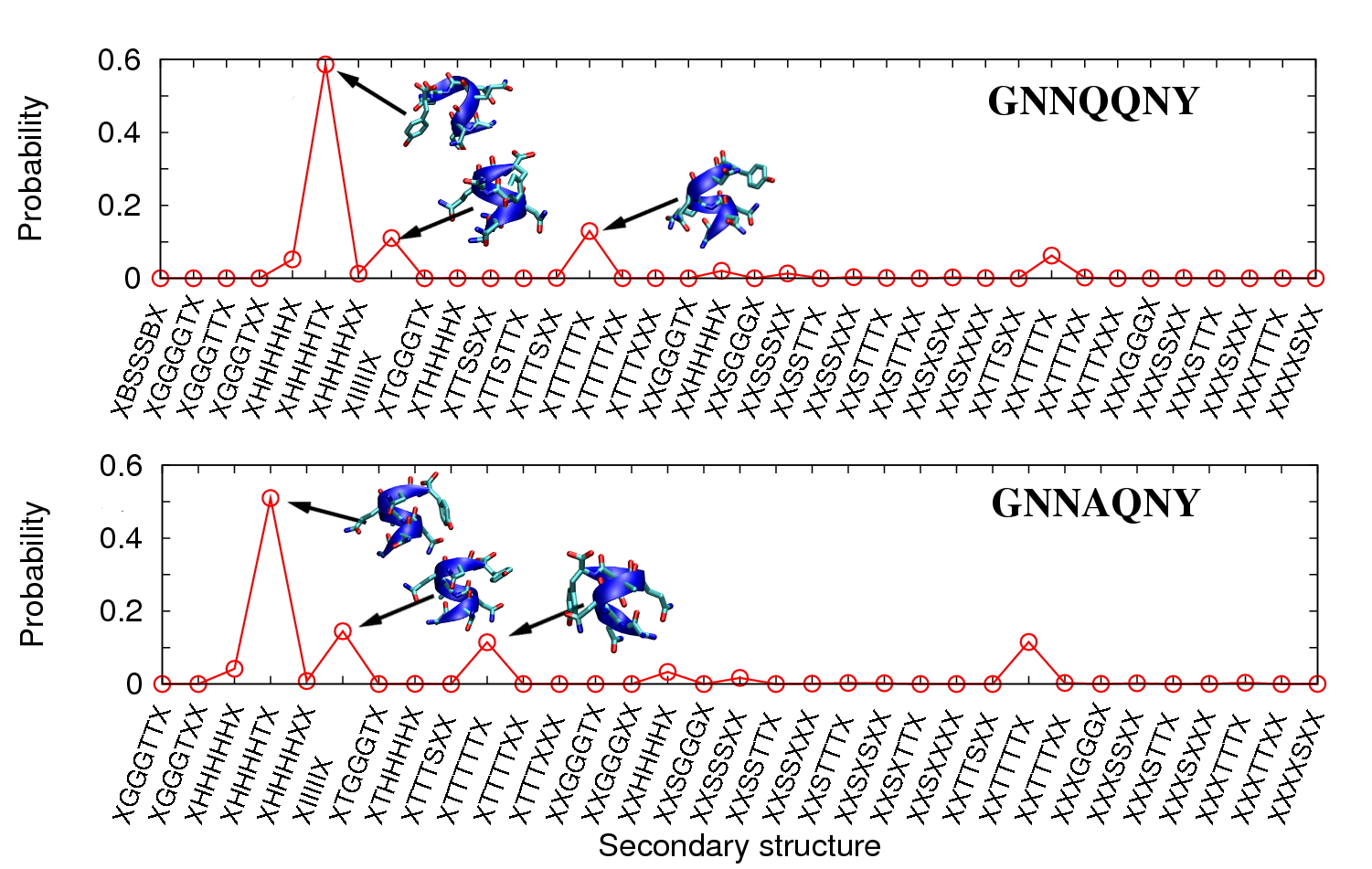

Supplement: Figures S9 — Secondary structure distribution of GNNQQNY and GNNAQNY monomer. After completing REMD simulation, each snapshot at 300 K were analyzed using the DSSP algorithm. The i-th character in the name of each conformation represents the secondary structure of the corresponding amino acid; X: unstructured, B: β-bridge, S: bend, G: 3-helix, T: hydrogen bonded turn, H: α-helix, and I: π-helix. (4.28 MB TIF) [file pcbi.1000492.s009.tif]
