# Supplementary material for: Thermodynamic Selection of Steric Zipper Patterns in the Amyloid Cross-β Spine
Source: PLoS Comput Biol. 2009 Sep 4;5(9):e1000492. doi: 10.1371/journal.pcbi.1000492 (PMC2723932; doi:10.1371/journal.pcbi.1000492)
Supplement: Table S1 — Correspondence between candidate patterns tested and eight steric-zipper classes proposed by Sawaya, et al. [27]. (0.01 MB PDF) [file pcbi.1000492.s010.pdf]

Parallel		Anti-parallel	
BBA and FFA	class 1	AregBB and AregFF	class 5
FBP	class 2	AregFB	class 6
BBP and FFP	class 3	AinvP	class 7
FBA	class 4	AinvA	class 8
