# Supplementary material for: Thermodynamic Selection of Steric Zipper Patterns in the Amyloid Cross-β Spine
Source: PLoS Comput Biol. 2009 Sep 4;5(9):e1000492. doi: 10.1371/journal.pcbi.1000492 (PMC2723932; doi:10.1371/journal.pcbi.1000492)
Supplement: Table S2 — Decomposition of ΔGbind of NNQQ bilayers. Native-like patterns are marked in bold. The energy unit is in kcal/(mol peptide) (0.02 MB PDF) [file pcbi.1000492.s011.pdf]

$d = 4.85$ Å	ΔE_{intra}	ΔE_{vdW}	ΔE_{elec}	ΔG_{hp}	ΔG_{screen}	ΔG_{NB}	$-T\Delta S_{vib}$	ΔG_{bind}
FBP1	2.90	-20.12	161.55	-9.84	-162.23	-27.23	2.74	-2.24
FBP2	2.02	-17.74	14.41	-8.55	-14.73	-24.02	1.52	-0.25
**FBA1**	3.04	-20.35	-29.20	-9.89	26.39	-29.97	1.56	-6.16
FBA2	2.12	-18.53	-27.11	-9.19	25.60	-26.88	1.41	-3.22
BBP	1.32	-16.99	65.31	-8.46	-65.59	-24.40	0.69	-1.46
BBA1	3.10	-20.42	-13.72	-9.68	12.80	-27.67	2.42	-3.00
BBA2	3.00	-20.60	-4.99	-9.73	4.48	-27.63	0.84	-4.54
FFP	3.11	-19.74	144.52	-9.59	-143.85	-25.55	3.42	0.12
FFA1	2.75	-20.20	-52.34 -	10.05	48.06	-31.57	2.50	-6.82
FFA2	2.52	-17.77	17.44	-8.48	-18.40	-24.71	1.61	-0.85

$d = 4.92$ Å	ΔE_{intra}	ΔE_{vdW}	ΔE_{elec}	ΔG_{hp}	ΔG_{screen}	ΔG_{NB}	$-T\Delta S_{vib}$	ΔG_{bind}
FBP1	2.38	-21.13	164.21	-9.91	-163.39	-27.84	2.65	-2.94
FBP2	1.13	-17.89	46.06	-8.59	-45.84	-25.13	1.62	-1.26
FBA1	2.62	-20.86	-17.53	-9.86	15.99	-29.64	2.17	-5.22
FBA2	3.34	-21.28	-18.89	-10.13	18.19	-28.77	2.79	-3.73
BBP1	1.85	-18.08	42.56	-8.78	-42.70	-25.14	2.24	-0.65
**BBA1**	2.30	-21.32	-11.67	-9.99	10.98	-29.69	3.09	-4.35
BBA2	3.01	-21.82	3.00	-10.01	-2.66	-28.48	1.99	-4.24
FFP1	0.80	-14.97	49.51	-6.97	-48.72	-20.35	0.26	2.16
**FFA1**	2.47	-20.46	-33.60	-9.84	31.17	-30.26	2.78	-5.23
FFA2	2.09	-16.76	-20.04	-8.25	18.57	-24.39	2.00	-0.14
