# Supplementary material for: Thermodynamic Selection of Steric Zipper Patterns in the Amyloid Cross-β Spine
Source: PLoS Comput Biol. 2009 Sep 4;5(9):e1000492. doi: 10.1371/journal.pcbi.1000492 (PMC2723932; doi:10.1371/journal.pcbi.1000492)
Supplement: Table S3 — Decomposition of ΔGbind of VEALYL bilayers. Native-like pattern is marked in bold. (0.02 MB PDF) [file pcbi.1000492.s012.pdf]

$d = 4.805$ Å	ΔE_{intra}	ΔE_{vdW}	ΔE_{elec}	ΔG_{hp}	ΔG_{screen}	ΔG_{NB}	$-T\Delta S_{vib}$	ΔG_{bind}
AinvA	-0.15	-23.29	32.33	-10.97	-28.44	-30.37	-2.66	-9.77
AinvP1	-0.63	-22.68	53.63	-10.57	-47.56	-27.18	-3.26	-7.71
**AinvP2**	1.07	-24.19	6.53	-11.86	-5.56	-35.08	-2.17	-12.76
Areg1BB	0.82	-21.69	4.51	-10.14	-4.24	-31.56	-4.43	-11.76
Areg1FB	-1.11	-23.18	47.99	-10.51	-43.13	-28.83	-3.57	-10.09
Areg1FF	-1.25	-22.83	22.27	-10.25	-16.80	-27.61	-4.50	-9.97
Areg2BB	-0.70	-22.25	63.94	-10.75	-56.53	-25.59	-1.58	-4.46
Areg2FB	0.27	-23.22	72.89	-10.94	-66.07	-27.34	-2.70	-6.28
Areg2FF	2.24	-22.35	81.59	-10.79	-81.06	-32.61	-3.20	-10.15
