# Supplementary material for: Thermodynamic Selection of Steric Zipper Patterns in the Amyloid Cross-β Spine
Source: PLoS Comput Biol. 2009 Sep 4;5(9):e1000492. doi: 10.1371/journal.pcbi.1000492 (PMC2723932; doi:10.1371/journal.pcbi.1000492)
Supplement: Table S4 — Decomposition of ΔGbind of KLVFFAE bilayers. The most stable (possibly native-like) structures are marked in bold. Selected configurations in pH 7.0 were further simulated with larged system sizes (20 peptides) and corresponding energy values are in parentheses. (0.02 MB PDF) [file pcbi.1000492.s013.pdf]

pH 7.0	ΔE_{intra}	ΔE_{vdW}	ΔE_{elec}	ΔG_{hp}	ΔG_{screen}	ΔG_{NB}	$-T\Delta S_{vib}$	ΔG_{bind}
Ainv1A	6.21	-31.16	-95.40	-12.23	86.65	-45.94	-10.84	-32.62
Ainv1P1	7.28	-32.26	-101.28	-12.98	90.63	-48.60	-10.34	-34.78
Ainv1P2	6.86	-32.59	-97.14	-12.73	87.48	-48.13	-10.36	-34.33
Ainv2A	5.86	-31.01	-109.95	-12.85	98.59	-49.36	-9.95	-35.15
Ainv2P1	6.08	-33.68	-90.06	-13.16	80.61	-50.21	-9.71	-35.76
	(8.25)	(-32.52)	(-107.46)	(-13.29)	(93.64)	(-51.47)		
Ainv2P2	6.25	-32.77	-97.39	-13.09	86.53	-50.48	-9.75	-36.07
	(8.16)	(-32.62)	(-106.59)	(-13.21)	(93.43)	(-50.82)		
**AregBB**	7.34	-31.01	-139.32	-13.07	126.25	-49.80	-11.06	-36.70
AregFB	6.53	-33.18	-130.51	-13.98	120.11	-51.02	-9.56	-36.42
**AregFF**	8.54	-28.44	-180.16	-13.38	161.93	-51.51	-10.16	-37.51
	(9.88)	(-30.22)	(-172.49)	(-13.38)	(155.79)	(-50.42)		
pH 2.0	ΔE_{intra}	ΔE_{vdW}	ΔE_{elec}	ΔG_{hp}	ΔG_{screen}	ΔG_{NB}	$-T\Delta S_{vib}$	ΔG_{bind}
**Ainv1A**	2.31	-27.71	-36.16	-9.86	41.08	-32.65	-13.49	-19.66
Ainv1P1	3.63	-26.31	-36.54	-9.19	41.77	-30.27	-14.06	-16.54
Ainv1P2	2.56	-26.47	-33.78	-9.05	38.53	-30.77	-13.76	-17.81
Ainv2A	2.80	-26.81	0.91	-9.00	5.98	-28.92	-13.03	-14.98
Ainv2P1	2.91	-27.73	0.27	-9.76	6.59	-30.63	-12.71	-16.27
Ainv2P2	2.88	-26.51	-35.05	-9.13	39.95	-30.74	-13.07	-16.77
AregBB	3.27	-28.33	-19.53	-10.34	26.37	-31.83	-13.56	-17.96
AregFB	3.47	-29.02	-10.59	-10.62	17.56	-32.67	-11.76	-16.79
AregFF	2.31	-27.57	9.19	-9.30	1.58	-26.10	-12.26	-11.89
