# Supplementary material for: Thermodynamic Selection of Steric Zipper Patterns in the Amyloid Cross-β Spine
Source: PLoS Comput Biol. 2009 Sep 4;5(9):e1000492. doi: 10.1371/journal.pcbi.1000492 (PMC2723932; doi:10.1371/journal.pcbi.1000492)
Supplement: Table S5 — Decomposition of ΔGbind of STVIIE bilayers. The most stable (possibly native- like) structure is marked in bold. (0.01 MB PDF) [file pcbi.1000492.s014.pdf]

	ΔE_{intra}	ΔE_{vdW}	ΔE_{elec}	ΔG_{hp}	ΔG_{screen}	ΔG_{NB}	$-T\Delta S_{vib}$	ΔG_{bind}
AinvA	11.40	-23.58	22.49	-7.99	-15.03	-24.11	8.69	19.45
AinvP1	18.04	-24.33	20.47	-8.57	-14.99	-27.42	8.88	24.38
AinvP2	15.96	-25.61	46.32	-8.55	-37.40	-25.24	9.04	24.02
Areg1BB	0.83	-21.10	78.53	-8.07	-73.92	-24.56	-4.85	-5.13
Areg1FB	-0.20	-18.72	79.17	-6.87	-74.41	-20.83	-4.88	2.68
Areg1FF	0.38	-15.61	59.10	-6.12	-55.07	-17.70	-3.71	3.52
Areg2BB	-0.98	-15.74	65.80	-5.07	-56.28	-11.29	-4.83	6.30
Areg2FB	-0.70	-17.33	66.60	-6.18	-59.27	-16.18	-4.86	2.00
**Areg2FF**	1.60	-23.39	36.54	-8.94	-32.89	-28.68	-3.44	-7.13
